# Manipulation of mitochondrial poly(A) polymerase family proteins in *Trypanosoma brucei* impacts mRNA termini processing

**DOI:** 10.3389/fpara.2023.1298561

**Published:** 2024-01-11

**Authors:** Clara M. Smoniewski, Poorya Mirzavand Borujeni, Marshall Hampton, Austin Petersen, Sean P. Faacks, Reza Salavati, Sara L. Zimmer

**Affiliations:** ^1^ Department of Biomedical Sciences, University of Minnesota Medical School, Duluth, MN, United States; ^2^ Institute of Parasitology, McGill University, Montreal, QC, Canada; ^3^ Department of Mathematics and Statistics, University of Minnesota Duluth, Duluth, MN, United States; ^4^ Department of Biology, University of Minnesota Duluth, Duluth, MN, United States

**Keywords:** African trypanosomiasis, nucleotidyltransferases, site-directed mutagenesis, mRNA tails, non-coding RNA, arginine methylation, Hidden Markov modelling

## Abstract

RNA-specific nucleotidyltransferases (rNTrs) add nontemplated nucleotides to the 3^′^ end of RNA. Two noncanonical rNTRs that are thought to be poly(A) polymerases (PAPs) have been identified in the mitochondria of trypanosomes – KPAP1 and KPAP2. KPAP1 is the primary polymerase that adds adenines (As) to trypanosome mitochondrial mRNA 3^′^ tails, while KPAP2 is a non-essential putative polymerase whose role in the mitochondria is ambiguous. Here, we elucidate the effects of manipulations of KPAP1 and KPAP2 on the 5^′^ and 3^′^ termini of transcripts and their 3^′^ tails. Using glycerol gradients followed by immunoblotting, we present evidence that KPAP2 is found in protein complexes of up to about 1600 kDa. High-throughput sequencing of mRNA termini showed that KPAP2 overexpression subtly changes an edited transcript’s 3^′^ tails, though not in a way consistent with general PAP activity. Next, to identify possible roles of posttranslational modifications on KPAP1 regulation, we mutated two KPAP1 arginine methylation sites to either mimic methylation or hypomethylation. We assessed their effect on 3^′^ mRNA tail characteristics and found that the two mutants generally had opposing effects, though some of these were transcript-specific. We present results suggesting that while methylation increases KPAP1 substrate binding and/or initial nucleotide additions, unmethylated KPAP1is more processive. We also present a comprehensive review of UTR termini, and evidence that tail addition activity may change as mRNA editing is initiated. Together, this work furthers our understanding of the role of KPAP1 and KPAP2 on trypanosome mitochondrial mRNA 3^′^ tail addition, as well as provides more information on mRNA termini processing in general.

## 1 Introduction

**Figure 1:**
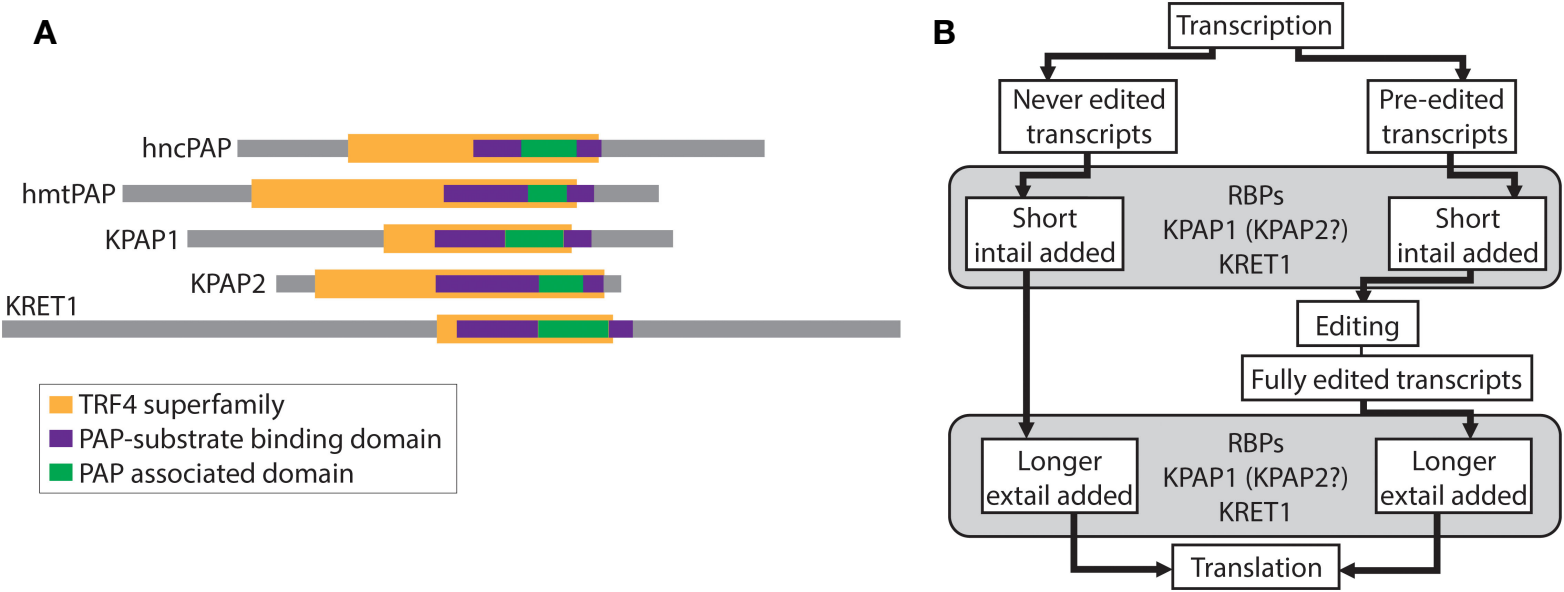
Relevant schematics of nucleotidyltransferases and the relationship between editing and tailing in trypanosomes. **(A)** Comparison of nucleotidyltransferases. Colored bars represent identified superfamilies and domains using TritrypDB, InterPro, and NCBI’s conserved domain search. hncPAP, human non-canonical PAP; hmtPAP, human mitochondrial PAP. **(B)** Schematic of the relationship between mRNA editing and mRNA 3^′^ tailing in trypanosomes. RBPs, RNA binding proteins; KPAP1, kinetoplast PAP1; KPAP2, kinetoplast PAP2; KRET1, kinetoplast RNA editing 3^′^ TUTase.

RNA-specific nucleotidyltransferases (rNTrs) are a part of the Pol beta-like nucleotidyltransferase (NT) superfamily that add ribonucleotides onto the 3^′^ ends of RNA strands in a nontemplated manner (Martin and Keller, 2007). This family is broken into canonical and non-canonical rNTrs based on both functional and structural characteristics. Canonical rNTrs are the nuclear poly(A) polymerases (PAPs) that act on pre-mRNA to polyadenylate the 3^′^ ends. The resulting 3^′^ poly(A) tails are known to affect the stability of mRNAs. Non-canonical rNTrs are more diverse and include cytosolic PAPs that act on various RNAs, mitochondrial PAPs, tRNA CCA-adding enzymes, and terminal uridylyl transferases (TUTases) (**Figure 1A**). Nucleotides added by non-canonical rNTrs have a diverse range of effects on RNA, from marking it for degradation (O’Hara et al., 1995) to stabilization (Decker and Parker, 1993).

TUTases were first isolated from cowpea leaves (*Vigna unguiculata*) (Zabel et al., 1981), but have been most extensively described in kinetoplastids (Aphasizhev et al., 2002; Aphasizhev et al., 2003). Kinetoplastids are a protozoan clade that includes infectious parasites such as *Trypanosoma brucei*, *T. cruzi*, and *Leishmania* spp., and are characterized by the presence of a single mitochondrion with a concatenated mitochondrial DNA and protein complex called a kinetoplast. TUTases are involved in a kinetoplastid-specific mitochondrial RNA editing process in which insertions and deletions of uridine (U) are required for some transcripts to be translatable. TUTases discovered in these organisms either add Us during the editing process (KRET2) or U-homopolymer to the 3^′^ ends of RNA (KRET1) that may also be acted upon by PAPs (Aphasizhev et al., 2003). U tails are added to small RNA editing templates called guide RNAs (gRNAs) and ribosomal RNA (rRNA), though their role is unclear (Aphasizheva and Aphasizhev, 2010). The precise effect of U-rich sequences is also not well understood when they are part of the heterogenous A/U tails that are added to the 3^′^ ends of mitochondrial mRNA.

Sharing some similar domains with KRET1 and 2 (**Figure 1A**), and initially thought to be TUTases themselves, are two kinetoplastid-specific PAP-family mitochondrial proteins - KPAP1 and KPAP2 (Kao and Read, 2007; Etheridge et al., 2008). These have been studied in the model organism *T. brucei*. KPAP1 is the primary polymerase that adds adenines (As) to mRNA 3^′^ tails and is part of the well-characterized polyadenylation complex (KPAC) (Etheridge et al., 2008; Aphasizheva et al., 2011; Zimmer et al., 2012). KPAP1 has also been found in purifications of a large ribonucleoprotein complex that includes RNA editing machinery (Aphasizheva et al., 2014). In contrast, KPAP2 is yet a putative polymerase that has not been identified in any protein complexes. Previous silencing of KPAP2 showed that while it was not essential, it did affect the tail length of one mitochondrial transcript (Kao and Read, 2007).

Heterogenous mitochondrial mRNA A/U tails are linked the unique kinetoplastid mRNA editing process (**Figure 1B**). In *T. brucei* 18 of the 20 mitochondrial transcripts are protein-coding and 12 of these require editing prior to translation. All 18 initial transcripts have short A/U tails added onto their 3^′^ end prior to or concurrent with editing initiation. These initially added tails (in-tails) are generally made up of homopolymers of As and Us with A-homopolymer regions predominating (Gazestani et al., 2016; Gazestani et al., 2018). In the current tail-addition model, after editing is complete, but prior to translation, the in-tails are extended into longer tails (ex-tails) (Etheridge et al., 2008; Aphasizheva et al., 2011). Ex-tails are identified by more frequent switching between A and U in the nucleotide chain than is typical of in-tails. A sub-population of never-edited transcripts also possesses ex-tails.

Trypanosome mitochondrial 3^′^ nonencoded tails have both life-stage and transcript-specific characteristics, such as their length profile and A/U composition, though how this specificity is achieved is unknown (Gazestani et al., 2016; Gazestani et al., 2018). Our previous work that examined homopolymer lengths in tails showed that there is potentially more variation in A-homopolymers than U-homopolymers (Hampton et al., 2021). This suggests that differential action of the KPAPs may be responsible for transcript and life stage specific tail characteristics. Changing protein levels, protein binding partner identities, and posttranslational modification are all possible methods of changing protein function (Goulah and Read, 2007; Bedford and Clarke, 2009; Gupta et al., 2013; Kale et al., 2018). While the effects of silencing KPAP2 have already been assessed (Kao and Read, 2007), overexpression of KPAP2 has not. Additionally, KPAP2 has not been found in any mitochondrial complexes, but its associations may provide clues to its role(s). In contrast, KPAP1 has been more extensively studied, with its protein binding partners and some posttranslational modifications already identified.

Here, we discuss the effects of manipulations of KPAP1 and KPAP2 on the 5^′^ and 3^′^ ends of transcripts, 5^′^ and 3^′^ untranslated regions (UTRs), and 3^′^ tails. 3^′^ and 5^′^ ends of transcripts were examined using circTAIL-seq, a circular RT-PCR and deep sequencing protocol. We present evidence that at least some KPAP2 is found complexed with other proteins and that overexpressing this PAP family member does subtly change a transcript’s 3^′^ tails, but not in a way consistent with general poly(A) polymerase activity. The effects of KPAP1 posttranslational modifications on transcripts’ tails characteristics was also assessed. We found that mutations mimicking posttranslational modifications did affect tail characteristics, some of which were transcript-specific, and that these modifications appear to decrease processivity of KPAP1. We also present a comprehensive review of UTR termini, and evidence that KRET1 and KPAP activity may change as mRNA editing is initiated.

## 2 Materials and methods

### 2.1 Plasmid construction and transfections

Oligonucleotides used for cloning are listed in **Supplementary Table S1**. The gene encoding KPAP2 (TriTrypDB Tb927.10.160.2) was inserted between the HindIII and BamHI sites in the pLEW100-LSH plasmid (Kafková et al., 2017) to generate pKPAP2-LSH for tetracycline (tet)-regulated expression of KPAP2 with a C-terminal linker-strep-his6 tag in 29-13 (Lister 427 strain expressing T7 polymerase and tetracycline repressor) procyclic form *T. brucei*. The gene encoding KPAP1 (TriTrypDB Tbg972.11.9070) was inserted between the HindIII and XhoI sites in the pLEW100-MHTAP plasmid (McAdams et al., 2015) to generate pKPAP1-MHT for tet-regulated expression of KPAP1 with a C-terminal myc-his6-TAP tag in 29-13 parasites. We used the same approach as Ringpis et al. (2011) for simultaneous depletion of endogenous KPAP1 and exogenous expression of WT or mutant KPAP1. The first 476 nucleotides of KPAP1 within the pKPAP1-MHT construct were removed by digestion with HindIII and PmlI and replaced with a gBlocks Gene Fragment (Integrated DNA Technologies) sequence designed by the RecodeTryps tool from the Read Lab (https://www.acsu.buffalo.edu/lread/tools.html). The sequence is shown in **Supplementary Table S1** and conserves the encoded amino acid sequence but contains silent mutations so that it is resistant to the tet-regulated RNAi that targets native KPAP1. An extra start codon in pLEW100 plasmids and the KPAP1 arginine methylation sites were mutated using the Q5 site-directed mutagenesis kit from New England Biolabs with primers in **Supplementary Table S1**. For tet-inducible RNAi of KPAP1, the first 471 nucleotides of KPAP1 were cloned into the p2T7-177 plasmid (Wickstead et al., 2002) between the BamHI and HindIII restriction sites to generate pKPAP1-RNAi.

### 2.2 Cell culture and growth curves

29-13 cells were grown in SDM-79 supplemented with G418 and hygromycin at 27°C in 5% CO_2_. Cells were a gift from Dr. Laurie Read, University at Buffalo in 2013. To generate tet-inducible cell lines, Not1-linearized plasmids were transfected into 29-13 cells, resulting in antibiotic-resistant polyclonal cultures. Puromycin was added to cells transfected with pKPAP1-MHT or pKPAP2-LSH. Phleomycin was added to cells transfected with pKPAP1-RNAi. Clones were obtained, when possible, by limiting dilution. For RNA and protein collection, cells were induced at 1x10^6^ cells/ml with tet every 24 hours and harvested at day 2 at about 1x10^7^ cells/ml. For growth curves, cells were started at 1x10^6^ cells/ml and diluted to 1x10^6^ cells/ml every two days. 2.5mg/ml tet was added to induced cells every 24 hours.

### 2.3 Cell fractionation

Cell fractionation was done as described in (Pelletier et al., 2007), omitting the Percoll density gradient. 2.5-5 x 10^8^ cells were harvested at log phase and pelleted. Cells were resuspended in ice-cold NET (0.15M NaCl, 0.1M EDTA, 10mM Tris-HCl pH 8.0), pelleted, and resuspended in DTE (1mM Tris-HCl pH 8.0, 1mM EDTA). Cells were lysed by passing through a 25G needle twice. 60% sucrose was immediately added and total protein was reserved. Lysed cells were pelleted and supernatant was reserved as non-organellar fraction. The pellet was resuspended in STM (250mM sucrose, 20mM Tris pH 8.0, 2mM MgCl2), DNase treated on ice for one hour and washed with STE (250mM sucrose, 20mM Tris pH 8.0, 10mM EDTA). The pellet was retained as the organellar fraction. Pellets were flash frozen in liquid nitrogen and stored in -80°C if further analysis was needed. After SDS-PAGE and transfer to nitrocellulose membrane, *T. brucei* antibodies (Vondrušková et al., 2005; Fisk et al., 2010) were used to probe for MRP2 as a mitochondrial control and PRMT1 as a cytosolic fraction control.

### 2.4 BN-PAGE

Organellar pellets from fractionation were thawed and lysed with 1% n-dodecyl-*β*D- maltoside (DDM) in 1x NativePAGE Sample Buffer (Invitrogen). After incubation for 15 minutes on ice, the sample was spun at 20,000g for 20 minutes at 4°C. Supernatant was collected, flash frozen, and stored at -80°C until use. Samples were prepared for electrophoresis with the addition of NativePAGE 5% G-250 Sample Additive (Invitrogen) to a final concentration of 0.25% before loading on a precast 10-well, NativePAGE Novex 3-12% of Bis-Tris Gels and run using the NativePAGE Bis-Tris Gel System Buffers and manufacturer’s protocol (Invitrogen). PVDF membrane was used for semi-dry transfer using NuPAGE Transfer Buffers (Invitrogen).

### 2.5 KPAP2 glycerol gradients

Mitochondria were isolated as in (Harris et al., 1990). Glycerol gradients were performed as in (Fisk et al., 2008) and 500*µ*l fractions were collected and analyzed by immunoblot. To calibrate fraction sedimentation, immunoblotting for KREPA2 and 3 was used to establish in which fractions the RECC (previously known as the 20S Editosome) appeared (Panigrahi et al., 2001).

### 2.6 Antibody purification and immunoblotting

Rabbit polyclonal serum was a gift from Dr. Laurie Read, University at Buffalo and raised against a 20 amino acid peptide of KPAP2 (C- ETKIE DVLNE LAKQG RLSSV) by Proteintech Group, Inc. (Chicago, IL). KPAP2 antibodies were affinity-purified from total serum with recombinant KPAP2 bound to nitrocellulose. To generate recombinant KPAP2 for this purpose, KPAP2 was expressed in *E. coli* by cloning the open reading frame of KPAP2 into pET-42a (Novagen) using NEBuilder assembly (New England BioLabs). IPTG induction was used to express KPAP2, the *E. coli* lysate was subjected to SDS-PAGE, transferred to nitrocellulose, and the band that obviously appeared upon induction at the appropriate size (contains rKPAP2 and *E. coli* proteins of the same size) was cut out. The membrane pieces were blocked overnight at 4°C with 5% milk in TBST and then incubated for 2 hours in 4ml serum. 0.2M glycine pH 2.8, 1mM EGTA was added and rocked for 3 minutes. pH was adjusted using 1M Tris pH 8.0, PBS was added to a final concentration of 1x, and 1% NaN_3_ was added to a final concentration of 0.02%. The purified KPAP2 antibody preparation was used in a 1:150 dilution overnight at 4°C, with goat anti-rabbit IgG as secondary antibody at a 1:15,000 dilution (Li-Cor Biosciences). The purified antisera recognized bands of just under 38 kDa, just over 38 kDa, and a doublet at about 60 kDa in lysates from 29-13 *T. brucei* (**Supplementary Figure S1A**). The bottom band was validated as being KPAP2 by its cross-reactivity with tagged overexpressed KPAP2-LSH detected by a commercial His antibody (Abcepta).

KPAP1 antibody was a gift from Inna Aphasizheva, Boston University Medical Campus and used at a 1:4,000 dilution overnight at 4°C, with goat anti-rabbit IgG as secondary antibody at a 1:15,000 dilution (Li-Cor Biosciences). KPAP1-MHT is about 55 kDa, while KPAP1 is about 75 kDa. A commercial antibody was used to detect the His tag (Abcepta). Monoclonal anti-*α*-tubulin antibody (Sigma-Aldrich) was used as a loading control.

For SDS-PAGE, cells were counted using a hemocytometer, pelleted, resuspended in PBS with SDS-PAGE sample buffer, and boiled before loading. Gels were transferred to nitrocellulose membranes using semi-dry transfer and 5% milk in TBST was used to block membranes prior to antibody incubations. A LiCor Odyssey Fc Imager was used to image and analyze gels and membranes using Image Studio Analysis software, version 5.2.5.

### 2.7 qRT-PCR

For qRT-PCR, total RNA was extracted from uninduced and induced cells as in (Shaw et al., 2016). Tet was added to induced cells every 24 hrs and collected on day 2. RNA was treated with the DNA-free kit (Invitrogen) to remove any residual DNA after which quality was verified by agarose electrophoresis. 4 µg of RNA was reverse transcribed using the TaqMan reverse transcription kit (Applied Biosystems). Resultant cDNA was amplified using Lightcycler 480 SYBR Green I Master Mix (Roche) and detected using a Lightcycler 480-II (Roche) and software version 1.5.1.62. Samples represent two biological replicates in duplicate wells. Primers used are listed in **Supplementary Table S1**. PFR was used for normalization.

### 2.8 CircTAIL-seq

CircTAIL-seq was performed as described in (Gazestani et al., 2016) and modified as described in (Smoniewski et al., 2023). Illumina primers and PCR conditions are listed in **Supplementary Table S2**. The University of Minnesota Genomics Center (UMGC) performed quality control on all libraries by assessing quality and quantity on an Agilent BioAnalyzer and performing KapaQC. One library, *CO1* KPAP1 WT -tet replicate 2, did not contain enough DNA and could not be sequenced. Amplicon libraries were then sequenced at UMGC on an Illumina MiSeq analyzer using the MiSeq V2 Chemistry 150 PE kit, acquiring 150 bp paired-end reads. One exception, *CYbp* KPAP1 cell lines replicate 1, were sequenced using MiSeq V2 Chemistry 250 PE kit. Eight libraries were run in one lane and sorted using barcodes. **Supplementary Table S3** lists the numbers of raw reads for each sequence file and, following processing, the number of merged reads utilized for downstream analysis of tails and UTR lengths. Processing was performed as in (Smoniewski et al., 2023). Read diversity was assessed and is presented in **Supplementary Table S4**.

### 2.9 Hidden Markov modelling

Hidden Markov modelling (HMM) was performed as described in (Gazestani et al., 2018) and (Hampton et al., 2021). To ensure consistency, models were trained hierarchically, with additional randomized states added one at a time after fully training the submodel, using the Baum-Welch algorithm.

### 2.10 Graphics

All graphs were made using R, version 4.2.1. Drawings were made using Adobe Illustrator version 27.0.0.

## 3 Results

Our aims were different for the two PAP-family proteins, KPAP2 and KPAP1. Relatively little is known about KPAP2, so we determined if KPAP2 was found in a complex and assessed the effect of its overexpression on mitochondrial mRNA tails. In contrast, KPAP1’s function and binding partners are already known, as are the general effects of manipulating KPAP1 expression. We therefore investigated protein arginine methylation as a possible regulation mechanism. Lastly, we evaluated both proteins’ effects on mRNA tails using hidden Markov modelling.

### 3.1 KPAP2

#### 3.1.1 KPAP2 is found both independently and within an unknown complex

**Figure 2:**
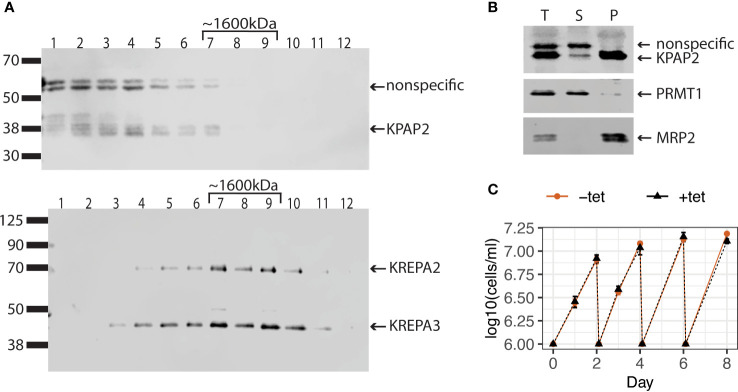
KPAP2 protein complex status and overexpression cell line characterization. **(A)** KPAP2 is found both independent, and in an unidentified protein complex. Immunoblotted fractions from a glycerol gradient of the mitochondrial fraction of the KPAP2 OE cell line. Numbers above gels represent the glycerol fraction from which the protein sample was taken. Membrane probed with the KPAP2 antibody (top) or antibodies to two proteins from the RNA-editing catalytic complex: KREPA2 and 3 (bottom). The experiment was performed in duplicate, with representative gels shown. **(B)** When induced with tetracycline, KPAP2 OE cell line overexpresses KPAP2, which is localized to the mitochondrion, as shown by cellular fractionation immunoblot. The supernatant (S) contains proteins present in the cytosol, while the pellet (P) contains proteins of organelles including the mitochondrion. Equivalent fractions of each were loaded. PRMT1, cytosolic control; MRP2, mitochondrial control. T, total protein. **(C)** Growth curves of induced with tetracycline (+tet) and uninduced (-tet) cells. Every two days cells are cut back to 1x10^6^ cells/ml. Error bars represent standard deviation of the mean (n = 3).

Unlike the situation for KPAP1, a plethora of pull-down and immunoprecipitation experiments have not identified KPAP2 as part of any known mitochondrial protein complex (Panigrahi et al., 2003; Etheridge et al., 2008; Aphasizheva et al., 2014). To determine if KPAP2 is functioning independently or within a complex, we attempted two methods to detect KPAP2 in its native associations in the mitochondrion. First, we performed blue native-polyacrylamide gel electrophoresis (BN-PAGE) on mitochondrial extract, identifying KPAP2 using a gene-specific antibody in a subsequent immunoblot (**Supplementary Figure S1B**). The antibody detected protein at a lower mass of less than 66 kDa, and at a higher mass between 1048 and 1236 kDa. 43 kDa is the estimated size of independent KPAP2, while the KPAP2 detected in higher density fractions may indicate that KPAP2 is present in complexes of various sizes. However, the purified KPAP2 antibody detects both KPAP2 and nonspecific proteins (**Supplementary Figure S1A**). Thus, to further elucidate this result, we separated protein complexes of the mitochondrial extract on a glycerol gradient (**Figure 2A**). By comparing the fractions containing KPAP2 (**Figure 2A**, top) to proteins from a complex of known mass (**Figure 2A**, bottom), we determined that KPAP2 was found in fractions ranging from the least dense (representing unbound proteins) to just under 1600kDa. While this result suggests that KPAP2 associates with other proteins, it is not clear which ones they may be, and multimers of KPAP2 are possible, as was found with KRET1 (Simpson et al., 2002). Additionally, KPAP1 has been found both unassociated and bound in a 1000 kDa complex that is believed to be a ribonucleoprotein assembly (Mesitov et al., 2019). As the samples in our KPAP2 association analysis were not RNase treated, nucleic acids may also contribute to observed KPAP2 complex sizes.

#### 3.1.2 KPAP2 overexpression does not affect cell growth

Though KPAP2 is present in both replicative *T. brucei* life stages, previous KPAP2 silencing did not affect parasite growth (Kao and Read, 2007). However, overexpression of enzymes acting on nucleic acids can be far more impactful (Aphasizheva and Aphasizhev, 2010; Zimmer et al., 2011). Thus, we modified procyclic stage parasites to inducibly overexpress KPAP2 tagged at the C-terminus with linker-strephistidine, referred to as the KPAP2 OE cell line. Utilizing a KPAP2 antibody, we determined that these cells express tagged KPAP2 at about eight-fold higher levels than untagged, native KPAP2 when induced with tetracycline (tet). To confirm that overexpressed KPAP2 was being directed to the mitochondria, we fractionated cells so that the supernatant contained cytosolic proteins and the pellet contained organellar, including mitochondrial, proteins (**Figure 2B**). Most of the overexpressed KPAP2 was found in the pellet fraction, suggesting mitochondrial localization. Similar to silencing, KPAP2 overexpression does not substantially impact cell growth (**Figure 2C**).

#### 3.1.3 KPAP2 overexpression generally does not change termini processing, but does change A composition of ex-tails in the edited version of the *A6* transcript

**Figure 3:**
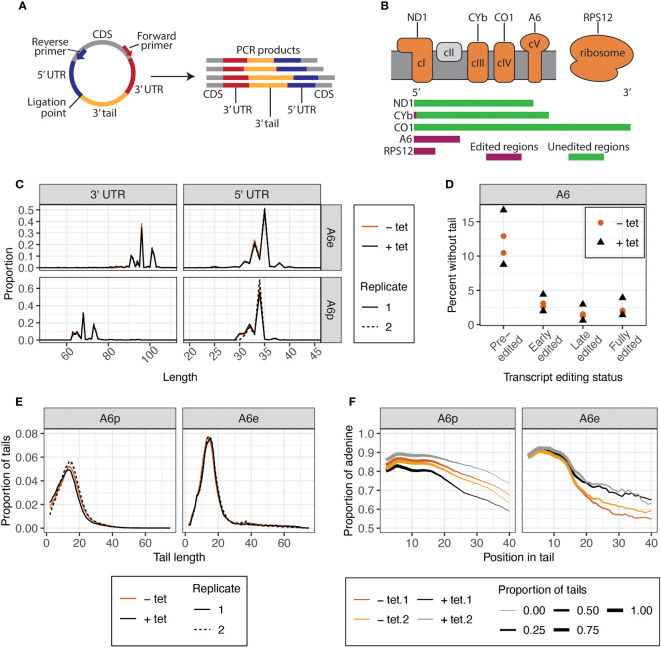
CircTAIL-seq is used to investigate 3^′^ tails and 3^′^ and 5^′^ ends of mitochondrially encoded transcripts. **(A)** CircTAIL-seq primer placement on *in vitro* circularized RNA, and PCR-based library generated in the utilization of these primers. CDS, coding sequence. Not to scale. **(B)** Top: The electron transport chain and mitochondrial ribosome with transcripts utilized in this study shown above the complexes of which they are a part. Complex II in grey does not have any subunits encoded in the mitochondrial genome. Bottom: Relative length and editing status of transcripts used in this study. The length of each transcript is proportional to the relative length of the mRNA prior to editing. Green, regions that do not require editing prior to translation; maroon, regions that require editing prior to translation. *ND1*, NADH dehydrogenase subunit 1; *CYb*, cytochrome b; *CO1*, cytochrome c oxidase subunit 1; *A6*, ATP synthase subunit 6; *RPS12*, ribosomal protein S12. **(C)** 3^′^ (left) and 5^′^ (right) UTR lengths of *A6* transcripts for KPAP2 OE cell line. **(D)** Percent of reads that do not possess untemplated A or U additions between the ligated ends of the 3^′^ and 5^′^ UTRs of *A6* transcripts at various editing stages in KPAP2 OE cell line. Experiment done in duplicate. Each circle or triangle represents one replicate. **(E)** Population density curves of tail lengths for *A6* transcripts in KPAP2 OE cell line. **(F)** Population density curves of proportions of nucleotides that are adenine at each position along tails for *A6* transcripts in KPAP2 OE cell line. The thickness of the line represents the proportion of total tails analyzed that are long enough to contribute to the data at each nucleotide position. As the lines become thinner, the proportion of tails that are available to contribute to the adenine content data approaches zero because there are very few long tails. Data is shown starting from the second nucleotide in tails to account for ambiguity in tail cutoff determination. Cells expressing KPAP2 at normal levels are uninduced (-tet, orange) and cells OE KPAP2 are induced with tetracycline (+tet, black). *A6*e, fully-edited *A6* transcripts defined by a fully-edited canonical coding sequence including the start codon; *A6*p, pre-edited *A6* transcripts with no evidence of editing initiation. A and B are modified from Smoniewski et al. (2023).

Despite lacking a growth phenotype, KPAP2 overexpression may affect mitochondrial termini or mRNA tails as was found for tail composition in a previous study of silenced KPAP2 (Kao and Read, 2007). To determine if this is the case, we analyzed tails with circTAIL-seq, which utilizes circular RT-PCR followed by Illumina sequencing of transcripts from the 3^′^ end, through the 3^′^ tail, to the 5^′^, end as described in (Gazestani et al., 2016) (**Figure 3A**). Our mRNA target of study was pre-edited and edited *A6*, which codes for a subunit of complex V of the electron transport chain (**Figure 3B**). *A6* tails have not been previously investigated under KPAP2 silencing. CircTAIL-seq primers spanned regions of the 3^′^ and 5^′^ ends that undergo RNA editing. Different primers were designed to bind to pre-edited and edited transcripts. Sequenced circTAIL-seq libraries were aligned to 3^′^ and 5^′^ termini templates. A portion of reads had a 3^′^ UTR directly ligated to 5^′^ UTR, and this was defined as a tailless read. All nontemplated nucleotides between the ligated 3^′^ and 5^′^ ends were defined as 3^′^ nonencoded tail sequence for that read. Processed circTAIL-seq reads were partitioned into their various segments: 3^′^ and 5^′^ untranslated regions (UTRs), and tails. For each transcript, these three mRNA elements were compared at the population level between the uninduced and induced KPAP2 OE cell line. As expected, overexpression of KPAP2, with its likely nucleotide-transferring activity, did not alter UTR lengths (**Figure 3C**). Regardless of KPAP2 expression, we did find that the 3^′^ UTR of *A6* is longer in edited transcripts relative to the UTRs on the pre-edited population of the same mRNA (**Figure 3C**). While the predominant terminal nucleotides are identical in both populations, additional uridines inserted by U-indel editing result in a longer 3^′^ UTR.

In addition to distinguishing tails from UTRs, evaluation of KPAP2 terminal ends yielded a process for categorizing the editing stage of sequence reads. Forward primers to capture termini and tails of pre-edited transcripts were placed proximal to stop codons, which resulted in the capture of reads from transcripts in which editing had initiated in its 3^′^ UTR in the pre-edited *A6* library, as well as completely pre-edited reads. Given the directionality of U-indel editing (3^′^ to 5^′^), this result is unsurprising. We separated these reads from the completely pre-edited reads and labelled them “early-edited” reads. Similarly, some reads from *A6* libraries generated with primers binding to fully-edited sequences were still not completely edited at the far 5^′^ end of transcripts. We separated these partially edited reads from the fully-edited reads and named them “late-edited” reads. In this way, we can divide libraries into reads from transcripts of four different editing states ranging from pre-initiation of editing to a fully-edited and translatable transcript. Numbers of reads partitioned into each category are found in **Supplementary Table S5**. This additional library partitioning allows insight into whether tails drive or are impacted by the initiation or progression of editing and is utilized in the rest of the study.

Some pre-edited transcripts will be captured prior to tails addition, but we expect the percentage of untailed transcripts to almost disappear in reads captured from transcripts where editing has initiated, as tails are thought to be essential for editing initiation (Etheridge et al., 2008; Zhang et al., 2017). Further, tails with more editing have been increasingly exposed to tail addition enzymes. Our data confirm this for all cell lines and transcripts that we examined (**Figure 3D**). We also noted that KPAP2 overexpression did not affect the percentage of reads without tails at any stage of editing (**Figure 3D**).

However, the most likely effect of KPAP2 overexpression would be changes to the characteristics of the 3^′^ tails themselves. We therefore measured mRNA tail length profiles for *A6* transcripts (**Figure 3E**) in cells with normal and high KPAP2 levels. There were no differences between the tails on pre-edited and early-edited or the fully-edited and late-edited transcripts, so tail metrics of only fully pre- or edited transcripts are shown. KPAP2 overexpression did not change 3^′^ mRNA tail lengths. Previous RNAi silencing of KPAP2 showed increased percentages of U in pre-edited *RPS12* tails but no overall length differences (Kao and Read, 2007), so we next examined A content of tails. While we saw no difference in A content of pre-edited *A6* tails, we did see A content of edited *A6* tails increase upon KPAP2 was overexpression (**Figure 3F**), especially on tails longer than 15 nt. However, given the low numbers of longer tails, we cannot exclude the possibility that the differences are the result of random chance. The subtlety of this phenotype suggests that KPAP2 might not play a sufficiently extensive role in tail addition to warrant further exploration. We thus turned our attention to the main A-adding enzyme KPAP1.

### 3.2 KPAP1

#### 3.2.1 KPAP1 mutated at two arginine methylation sites is expressed in modified cell lines

Two arginine methylation sites have been identified on KPAP1 that are monomethylated in both life stages. Which of the two sites are methylated at any given time is unknown as they were identified in a proteome-wide study (Lott et al., 2013). At any point, there is likely to be a mixed population of methylated, unmethylated, or partially methylated (one out of the two sites) KPAP1 proteins in the mitochondrion. Since we observed no indication that KPAP2 activity contributes to prominent transcript-specific and life stage-specific differences between tail populations, the mechanism by which the parasite may impart these differences may be by modulating the activity of KPAP1 on specific transcript termini. Such modulation of function can be imparted by post-translational protein modifications such as the two arginine methylation sites on KPAP1.

**Figure 4:**
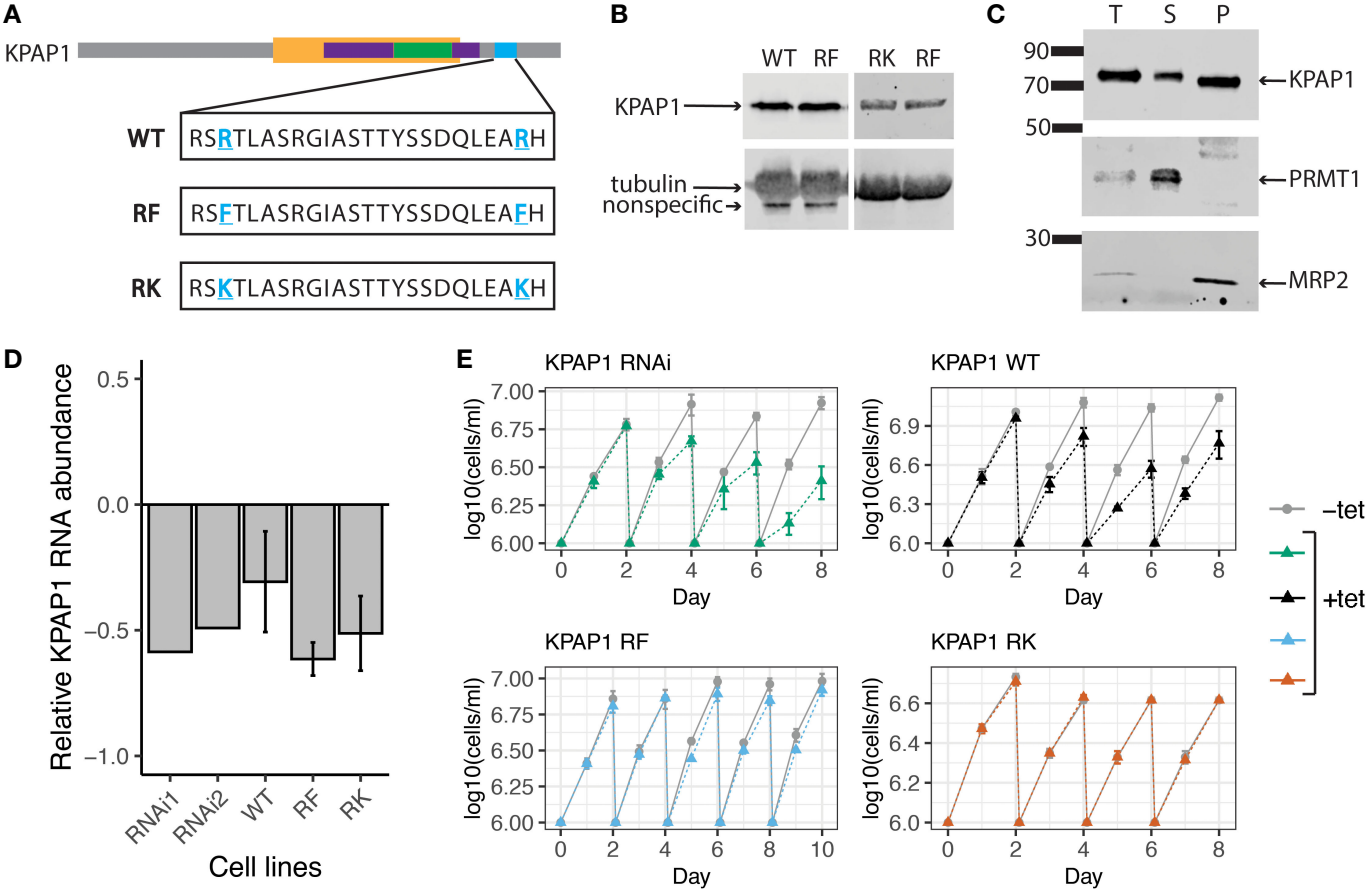
KPAP1 cell lines overexpress tagged KPAP1 and silence native KPAP1 when induced with tetracycline. **(A)** Schematic of the three versions of KPAP1 used in the KPAP1 cell lines. Bolded, underlined, blue residues indicate identified arginine methylation sites that have been subsequently mutated to indicated amino acids. WT, wildtype; RF, arginine to phenylalanine (methylmimic); RK, arginine to lysine (methylmutant). TRF4 superfamily, yellow; PAP-substrate binding domain, purple; PAP associated domain, green; location of arginine methylation, blue. **(B)** Immunoblotting shows similar levels of tagged KPAP1 overexpression in cell lines at day 2 following induction by tetracycline as detected by histidine antibody. Tubulin used as a loading control. **(C)** Cellular fractionation immunoblot. The supernatant (S) contains proteins present in the cytosol, while the pellet (P) contains organelles such as the mitochondrion. PRMT1 is used as a cytosolic control, MRP2 is used as a mitochondrial control. T, total protein. **(D)** RT-qPCR results show a decrease in native KPAP1 expression in induced cell (+tet) compared to uninduced cell (-tet) at day 2 following tetracycline induction. RNAi1 and 2 represent two different clones. Error bars represent standard deviation of the mean. **(E)** Cell growth defects associated with KPAP1 silencing are rescued by mutant KPAP1 overexpression, but not by WT KPAP1 overexpression. KPAP1 RNAi cell line silences expression of KPAP1 when induced with tetracycline. KPAP1 WT cell line when induced silences native KPAP1 while an exogenous wildtype version of KPAP1 is overexpressed. KPAP1 RF (methylmimic) and KPAP1 RK (hypomethyl) cell lines, when induced, silences native KPAP1 while an exogenous mutated version of KPAP1 is overexpressed. KPAP1 RF growth curve was extended to day 10 because of the slight difference in growth at day 8. Error bars represent standard deviation. Cells are kept in log phase growth by diluting them every two days to 1x10^6^ cells/ml. Experiments were done in triplicate. Uninduced, -tet; induced, +tet.

To determine if KPAP1 arginine methylation affects 3^′^ tail characteristics, we created three cell lines in which the native KPAP1 was silenced while one of three different versions of KPAP1 was expressed: WT, which expresses wildtype KPAP1 (addback); methylmimic or RF, in which the two KPAP1 arginine methylation sites were mutated to phenylalanine; and hypomethyl or RK, in which the same two arginine methylation sites were mutated to lysine (**Figure 4A**). In the KPAP1 WT cell line, KPAP1 arginine residues will be methylated/demethylated as normal. In the methylmimic, the bulkiness of phenylalanine (F) mimics methylation, so this cell line represents constant methylation of the KPAP1 arginine methylation sites. Correspondingly, lysine (K) in the hypomethyl cell line is positively charged like arginine yet cannot be methylated by arginine methyltransferases. The lysine residues act as constantly unmethylated arginine. In experiments, we compare the methylmimic, RF or hypomethyl, RK extremes to wildtype methylation (WT). All three versions of KPAP1 were also C-terminally tagged with a myc-histidine-tandem affinity purification (MHT) tag.

To ensure that we were comparing the effects of the KPAP1 arginine methylation site mutations independent of expression level, we verified that each version of KPAP1 was expressed similarly (**Figure 4B**). Densitometry analysis of KPAP1 identified on immunoblots analyzing the three induced cell lines revealed that KPAP1 is about 30 times more highly expressed than native KPAP1 in the KPAP1 WT cell line. Overexpression of KPAP1 is not a part of the experimental design but a byproduct of genetic tools available in *T. brucei* for inducible expression. Using cell fractionation, we also showed that the tagged KPAP1 was localized to the mitochondrion as expected (**Figure 4C**). Silencing of native KPAP1 was confirmed using RT-qPCR (**Figure 4D**). Though the amount of silencing of the native enzyme varied at the mRNA level between cell lines, these differences were likely insignificant given the level of exogenous KPAP1 overexpression.

KPAP1 depletion negatively affects cell growth (Etheridge et al., 2008). We confirmed this with our own native KPAP1 RNAi silencing scheme (**Figure 4E**). We then tested if any of the KPAP1 addbacks would rescue this phenotype. Surprisingly, overexpression of WT KPAP1 did not rescue the KPAP1 RNAi phenotype (**Figure 4E**, top left), yet methylmimic and hypomethyl KPAP1 overexpression did (**Figure 4E**, bottom row). We were then curious whether overexpression alone would affect cell growth. Growth curves of cell lines that only overexpressed a version of KPAP1 (WT, RF, or RK) without silencing the native KPAP1 showed that overexpression alone produces similar trends as when combined with KPAP1 RNAi. Thus, overexpression of WT KPAP1 was detrimental to cell growth but overexpression of either of the mutants (RF or RK) showed no change in growth when compared to uninduced cells (**Supplementary Figure S2**). Possibly, WT KPAP1 overexpression allows some atypical activity that cannot be supported by the parasite, while mutation of both arginine residues prevent this unwanted activity.

#### 3.2.2 Mutations to KPAP1 arginine methylation sites do not change termini processing but do affect tail addition initiation in a transcript-specific manner

To define possible impacts of KPAP1 arginine methylation on its function as a tail-adding polymerase, we examined mRNA 3^′^ and 5^′^ UTRs, and 3^′^ tails in all three cell lines. We first determined that, when uninduced, all cell lines, including the KPAP2 OE cell line, produced similar length tails with similar A compositions on a test transcript, *CO1* (**Supplementary Figure S3**). As tails were similar for all uninduced cells, we sequenced only the KPAP1 WT uninduced cell line as a control along with the three induced KPAP1 cell lines. As stated above, while overexpression of KPAP1 was not the goal, it was a byproduct of the available tools for inducible mutation in *T. brucei.* Therefore, before comparing termini generated in KPAP1 WT versus methylmimic and hypomethyl cell lines, we compared termini in cells expressing normal amounts of KPAP1 (uninduced, -tet) to cells that were overexpressing WT KPAP1 (induced, +tet). This positive control revealed the effect of overexpresison of the main poly(A) polymerase on mRNA termini.

One transcript from each mitochondrially-encoded electron transport chain complex was chosen for circTAIL-seq (*ND1*, *CYb*, *CO1*, *A6*), as well as a transcript encoding a ribosomal protein (*RPS12*) (**Figure 3B**). A variety of editing requirements were represented: transcripts that require extensive editing before translation (*A6* and *RPS12*), minimal editing (*CYb*), and those that are never-edited (*CO1* and *ND1*). Transcripts that require editing were separated into four groups as described above: pre-edited libraries were separated into pre-edited or early-edited reads, while edited libraries were broken into fully-edited and late-edited reads. Each of these four different editing stages was analyzed separately, except for *CYb* early-edited reads of which there were too few to analyze (**Supplementary Table S5**). This was most likely due to primer placement and the fact that a very small region of the gene is edited and is consistent with previous studies (Simpson et al., 2016; Carnes et al., 2017; Tylec et al., 2019).

**Figure 5:**
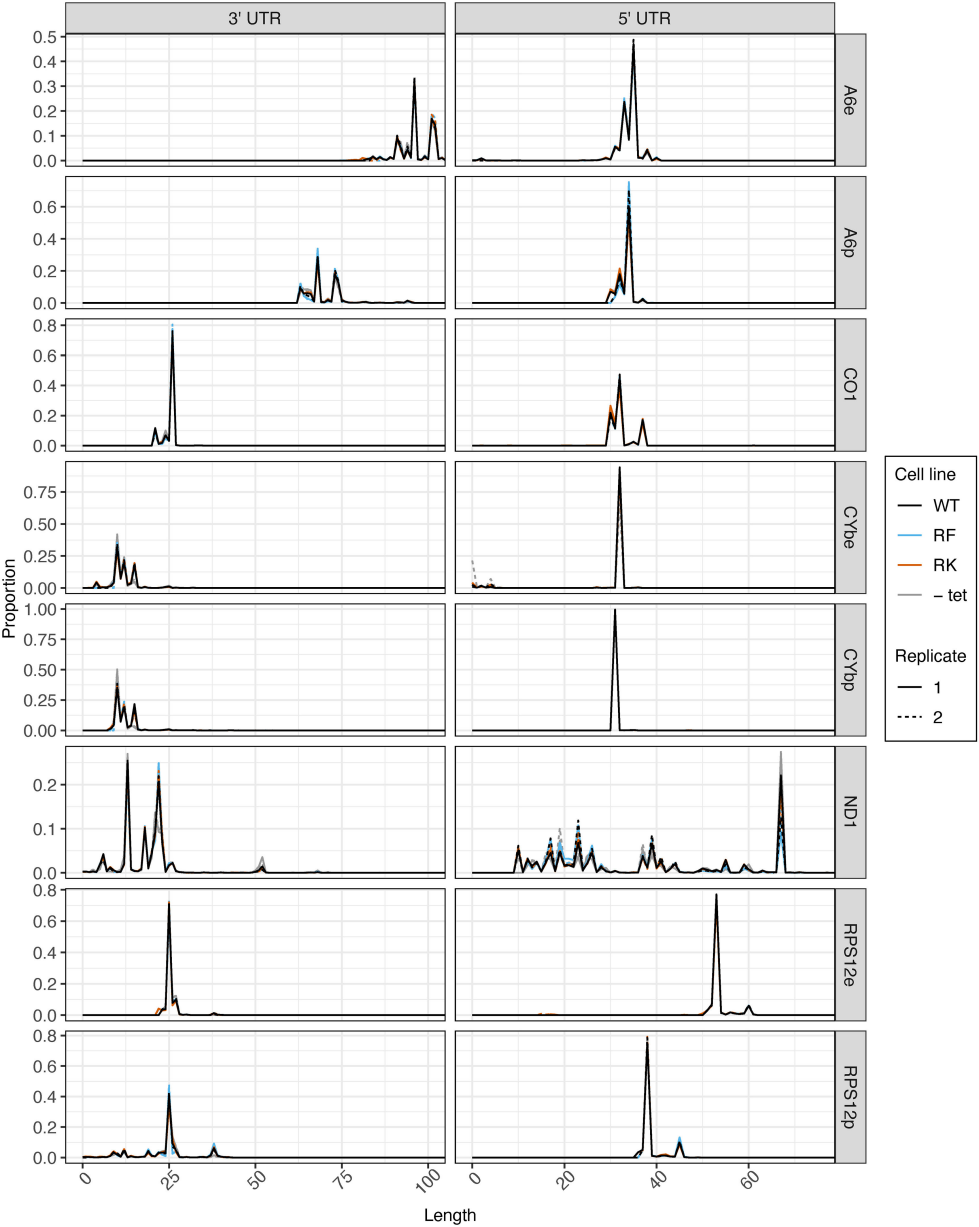
5^′^ UTR length is consistent between transcripts, and mutations at KPAP1 arginine methylation sites do not affect transcripts’ UTR processing. 3^′^ (left) and 5^′^ (right) UTR lengths of five mitochondrial transcripts names on the far right. e, fully-edited transcripts (i.e., start codon and all required coding region uridine insertions and deletions are present); p, pre-edited transcripts (i.e., no evidence of editing initiation). 5^′^ UTR lengths are counted so that the first nucleotide upstream of the adenine of the start codon is +1. 3^′^ UTR lengths are counted so that the last nucleotide after the stop codon is position +1. Partially-edited transcripts were not considered. There is only one replicate for -tet *CO1* as noted in the methods.

Prior to tail analysis, we assessed UTR lengths of all transcripts (**Figure 5**). 3^′^ and 5^′^ UTR processing was not affected by mutations to KPAP1 arginine methylation sites, as KPAP1 does not play a known role in determination of UTR length. However, this is the most comprehensive and high-throughput assessment of trypanosome mitochondrial UTR lengths currently available, so we utilized the data to determine population-level UTR length trends within and between transcripts. 3^′^ UTR lengths differed among transcripts, though those of *CO1* and *RPS12* are similar. For all transcripts besides *A6*, 3^′^ UTRs are about 25 nt or shorter. Interestingly, *A6* is also the only transcript in which editing in the 3^′^ UTR increases the 3^′^ UTR length substantially, by about 25 nt, as we also showed for *A6* derived from the KPAP2 OE cell line (**Figures 3C**, **5**, comparing *A6*e to *A6*p). In contrast to the more variable 3^′^ UTR lengths, 5^′^ UTR lengths of *A6*, *CO1*, *CYb*, and pre-edited *RPS12* are remarkably similar. Only *RPS12*’s 5^′^ UTR undergoes substantial editing to increase its length by about 15 nt. Finally, while the 3^′^ and 5^′^ UTRs of most transcripts have one or, at most, two common terminal positions, the *ND1* UTR length is extremely variable. In particular, *ND1*’s 5^′^ UTR has many shorter UTRs than the most common length of just under 70 nt. *ND1* is also the only transcript that we sequenced with a maxicircle loci that does not have another coding loci proximal to its 5^′^ end. In summary, 3^′^ and 5^′^ UTRs generally end at a single or very few nt positions, most 5^′^ UTR lengths are similar across the analyzed transcripts, and 3^′^ UTR lengths are more variable.

**Figure 6:**
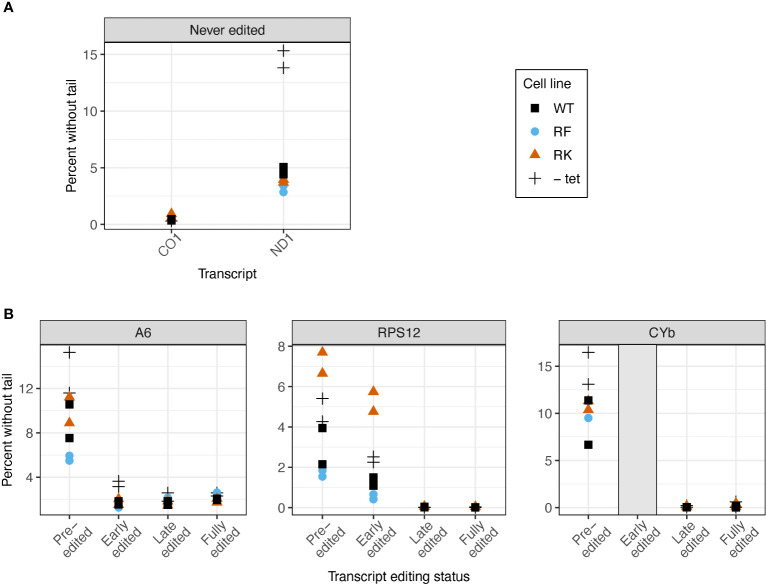
Mutations at the arginine methylation sites on KPAP1 affect likelihood of the presence of a tail on transcripts in two out of five transcripts tested. **(A)** Percent of reads that possess no untemplated A or U additions for two never-edited transcripts. **(B)** Percent of reads that possess no untemplated A or U additions for three transcripts at various stages of editing. Grey box for the early-edited *CYb*p tails indicates there were too few tails to analyze. Note scale differences for y-axes. Each experiment done in duplicate, represented by two of the same shape in each column.

We next examined whether KPAP1 manipulations affected the probability that transcripts are tailed at all. Two known polymerases add terminal nucleotides to mRNAs in the mitochondrion: the U-adding KRET1 that is part of the mitochondrial 3^′^ processome (MPsome), and A-adding KPAP1 as part of KPAC. Predictably, overexpression of WT KPAP1 in general leads to lower percentages of tail-less reads. However, when comparing 3’ ends of transcripts between cell lines with WT and mutant KPAP1, we found that KPAP1 manipulations had transcript-specific effects on the proportion of 3^′^ ends that possessed untemplated nucleotides. The finding was unrelated to whether the initial polymer string of the tails tended to be A (added by KPAC) or U (added by MPsome) within each population. Of the never-edited transcripts *CO1* and *ND1*, only *ND1* showed differences between the cell lines, because only *ND1* had appreciable numbers of tail-less transcripts (**Figure 6A**). The methylmimic had the fewest percent of *ND1* transcripts without tails, while the WT cell line showed the highest percent of tailless transcripts.

In reads that are edited, we examined percent of transcripts without tails as transcripts progress through the editing process. As expected, the percent of sequences lacking tails decreases to zero or nearly zero as editing is initiated, regardless of arginine methylation site mutation (**Figure 6B**). Only *RPS12* showed an effect of KPAP1 arginine methylation site mutations on the percentage of transcripts lacking tails. Of the pre-edited and early-edited *RPS12* reads, the hypomethyl (RK) cell line had more tailless transcripts than the KPAP1 WT cell line, while the methymimic (RF) cell line had slightly fewer transcripts without tails than the KPAP1 WT cell line. Thus, mutations to KPAP1’s arginine methylation sites affect tail-addition initiation in a transcript-specific manner, only affecting two out of the five transcripts tested, with the effect on the percentage of *ND1* tail-less reads being minor.

#### 3.2.3 Overexpression of wildtype KPAP1 does not increase tail length of transcripts at the population level, but mutations to KPAP1 arginine methylation sites do affect the length of tails

**Figure 7:**
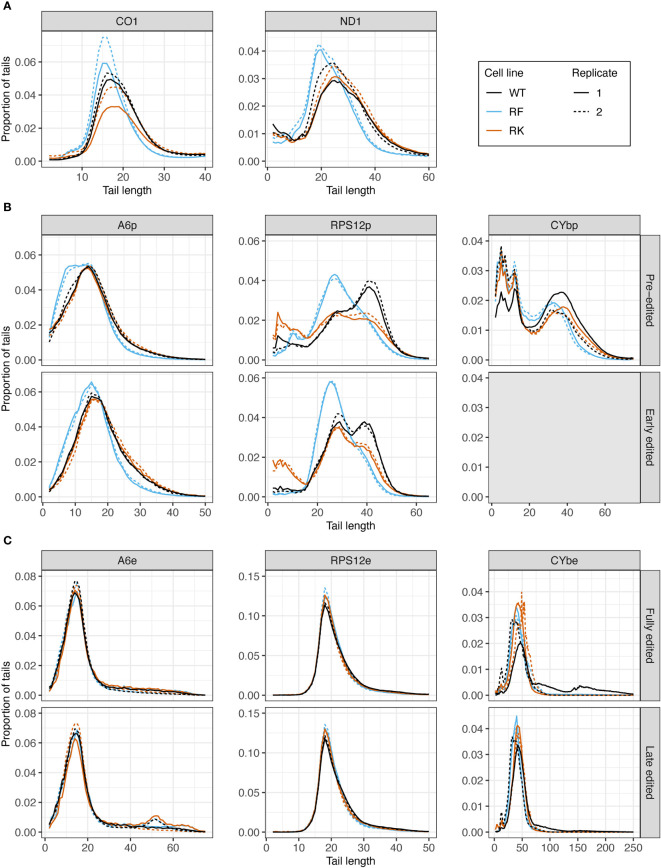
Mutations at the arginine methylation sites on KPAP1 affect the tail length of never-edited and pre-edited transcripts. **(A)** Population density curves of tail lengths for never-edited transcripts *CO1* and *ND1* zoomed in on the more abundant shorter tails, which are less than 60 nt. **(B)** Population density curves of tail lengths for pre-edited and early edited transcripts *A6*, *RPS12*, and *CYb*. Grey box for the early-edited *CYb*p tails indicates there were too few tails to analyze. **(C)** Population density curves of tail lengths for fully-edited and late edited transcripts *A6*, *RPS12*, and *CYb*. Reads are derived from primers designed to bind to either pre-edited transcripts (p) or edited transcripts (e).

Next, we assessed the effect of KPAP1 WT overexpression on tail length profiles. Similar to tails from KPAP2 OE cells, tails in +tet KPAP1 WT cells were not substantially longer than in normal cells (**Supplementary Figure S4**). *ND1* tails were an exception (**Supplementary Figure S4A**). This was in contrast with previous work indicated a shortening of tails on *CO1* and *RPS12* with silencing of KPAP1 (Etheridge et al., 2008) in gel electrophoresis studies. But this same paper found that even with increasing concentrations of recombinant protein, A tail addition stopped at about 20-25 nt. Interestingly, mutation of the KPAP1 arginine methylation sites did affect tail length in all five transcripts tested (**Figure 7**). Effects were seen in the shorter in-tails rather than the longer and more variable translation-related A/U ex-tails (**Figures 7**, **S5**). On never-edited transcripts the methylmimic produced in-tails that were shorter than those in the WT or hypomethyl mutant cell lines, while no differences in tail length of the longer ex-tails were seen (at least in the ex-tails we were able to capture with this method, **Supplementary Figure S5**). Looking across the entire population size range, the methylmimic produced shorter tail populations for all five transcripts while the hypomethyl cell line produced tails that were often quite similar to KPAP1 WT. Incidentally, while we obtained ex-tails in all the never-edited transcript libraries (**Supplementary Figure S5**), we rarely captured them in the fully-edited libraries and infrequently captured longer tails in the late-edited transcript libraries (**Figure 7C**).

#### 3.2.4 Overexpression of wildtype KPAP1 increases A composition of tails, while KPAP1 arginine methylation site mutation has transcript-specific effects

Tail nucleotide composition may also be impacted by differential KPAP1 activity. As we did with length, we first compared tail population A composition in uninduced, normal-behaving KPAP1 WT cells (-tet) to induced, overexpressing KPAP1 cells (+tet). We found that induced cells produced tails with higher A content than -tet cells, but that this increase manifested in different magnitudes and at different positions along the tails depending on the transcript population analyzed. Adenine content was higher throughout the tails of never-edited transcripts (**Supplementary Figure S6A**) (less so for *CO1*) while it was only higher in certain regions of the tail (at the population level) in transcripts that undergo editing (**Supplementary Figures S6B, C**). While pre-edited *CYb* and *RPS12* only showed increased A content at the beginning of tails in overexpressing cells, pre-edited *A6* showed increased A content throughout the length of the tail (**Supplementary Figure S6B**). These trends were also true for early-edited *A6* and *RPS12*. In contrast, fully-edited *A6* and *RPS12* tails only have increased A content as the tails approach 20 nt and longer, whereas fully-edited *CYb* shows increased A content only at the start of the tail (**Supplementary Figure S6C**). Although this trend in *CYb* fully-edited tails is also found in its late-edited tails, *A6* and *RPS12* late-edited tails showed no discernible trends. The tail position and transcript-specific effects of KPAP1 overexpression suggest that KPAP1 activity may be dependent on both the tail position at which As are added, and on the identity of the transcript on which it is adding a tail. Overall, the fact that overexpression of KPAP1 generally increases A composition of tails is expected as it is a poly(A) polymerase and thus is a good positive control.

**Figure 8:**
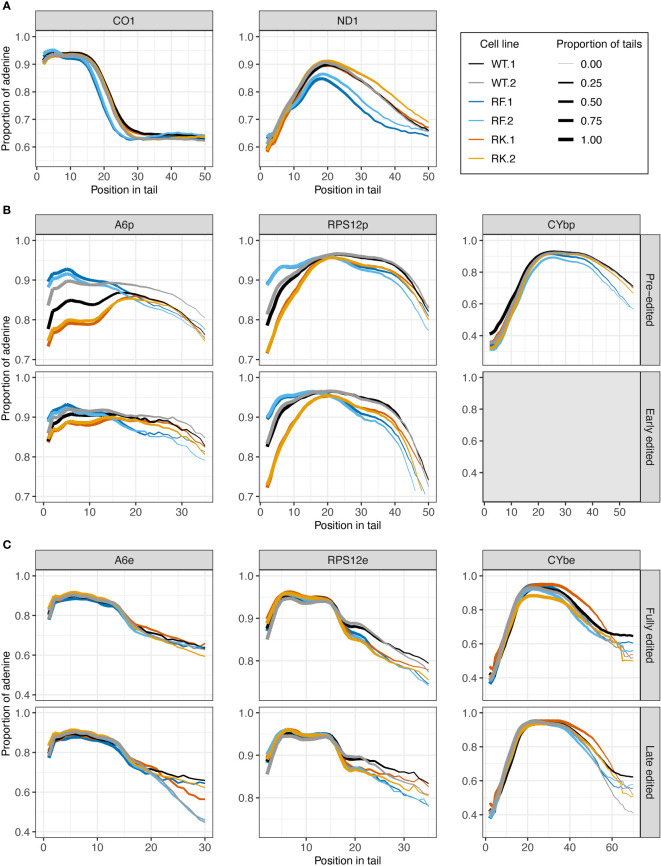
Mutations at the arginine methylation sites on KPAP1 affect the adenine composition of tails of never-edited and pre-edited transcripts. **(A)** Population density curves of proportion of adenine along tail for never-edited transcripts *CO1* and *ND1* zoomed in on the more abundant shorter tails, which are less than 60 nt. **(B)** Population density curves of proportion of adenine along tail for pre-edited and early-edited transcripts *A6*, *RPS12*, and *CYb*. Grey box for the early-edited *CYb*p tails indicates there were too few tails to analyze. **(C)** Population density curves of proportion of adenine along tail for fully-edited and late edited transcripts *A6*, *RPS12*, and *CYb*. Reads are derived from primers designed to bind to either pre-edited transcripts (p) or edited transcripts (e).

Impacts of KPAP1 methylation on nucleotide composition were next determined, utilizing among the WT, methylmimic, and hypomethyl cell lines (**Figure 8**). In never-edited transcripts *CO1* and *ND1*, and in pre-edited *CYb*, tails from WT and hypomethyl cell lines (in orange) have similar A content throughout the tail lengths, though in *ND1* tails, the hypomethyl cell line produced tails with fewer As at the beginning through about the tenth nucleotide position (**Figures 8A, B**). Pre- and early-edited *A6* tails showed a decrease in A composition in the hypomethyl cell line at the beginning of the tails relative to WT, while pre- and early-edited *RPS12* showed a decrease in A content throughout the tail length except around 20 nt (**Figure 8B**). In contrast, methylmimic cell lines (in blue) produced more As at the beginning of tails of all never-edited and pre-edited transcript tails except *CYb*, though pre-edited *A6* and *RPS12* showed the most dramatic increase in A content. After about the tenth nucleotide position never-edited *CO1* and *ND1* showed a substantial decrease in the proportion of As in the tails in the methylmimic, as did early-edited *A6* (after 15 nt), pre-edited *CYb* (after 30 nt), and all *RPS12* tails (after 20 nt). Similar to the tail length analysis, no effects of either KPAP1 mutation were observed in the nucleotide composition of edited transcript tails, except in *RPS12* in which both mutants show decreased A content after about the fifteenth nucleotide position when compared to WT (**Figure 8C**).

In summary, the most consistent differences in A composition between the cell lines appeared in the never-edited and pre-edited transcripts at the beginning of the tails, which were: (1) the increase in A composition for the methylmimic and (2) the decrease in A composition for the hypomethyl mutant. Also consistent across these same transcript groups is the decrease in A composition later in the tails for the methylmimic cell line.

### 3.3 Hidden Markov modelling — KPAP1 and KPAP2

**Figure 9:**
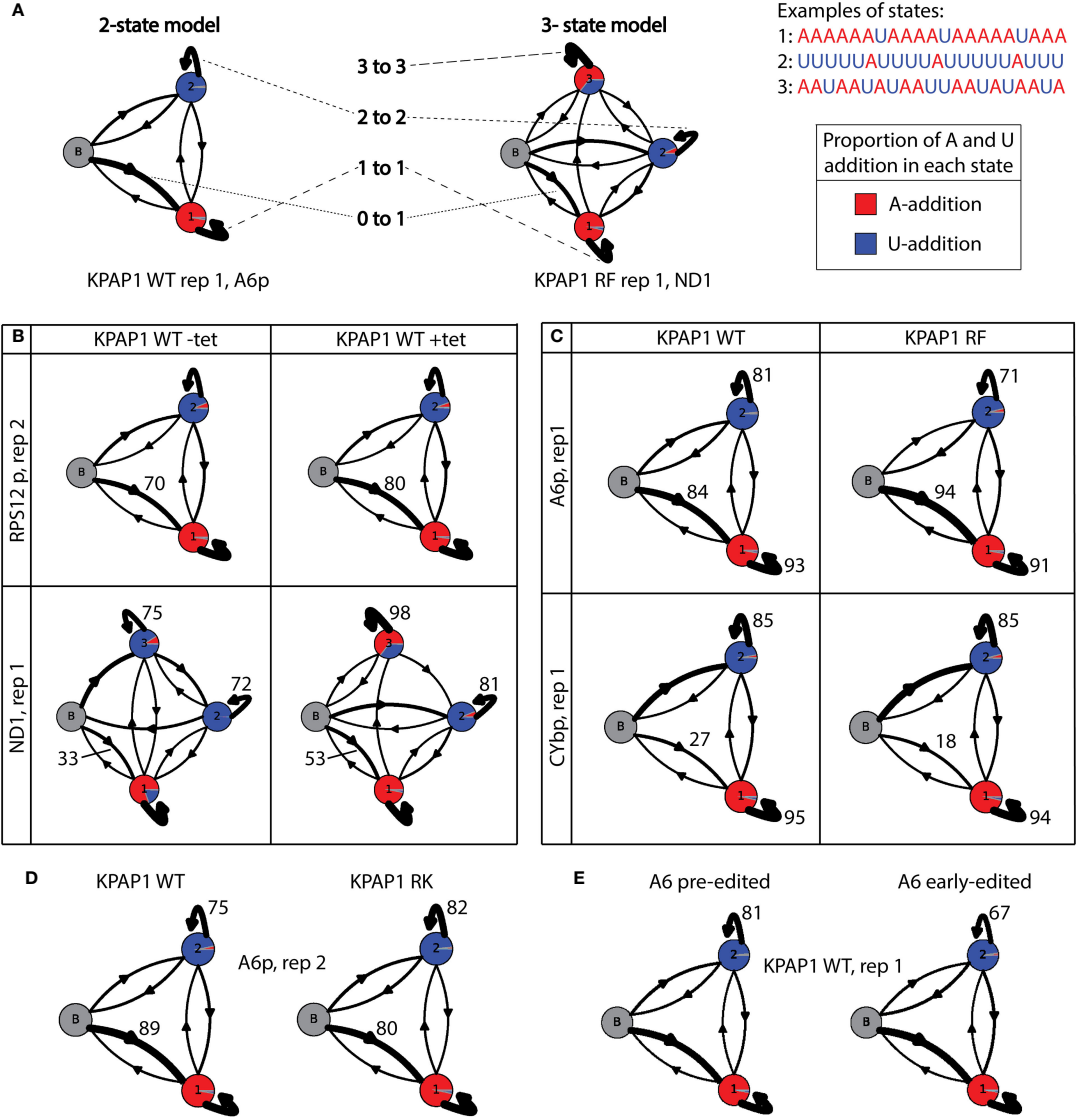
Hidden Markov modelling elucidates A- and U-addition characteristics. **(A)** Examples and explanations of 2-state and 3-state tail addition models. ‘N to n’ corresponds to numbers in **Supplementary Tables S6, S7**, and**S8**. Proportion of each circle colored is described as ‘A to n’ in **Supplementary Tables S6, S7**, and **S8**. Examples are derived from real data as indicated below each model. **(B)** Examples showing the differences between models derived from uninduced KPAP1 WT (-tet) cell lines to those that were induced (+tet). Of interest is the increase in tails that start in the primarily A-addition state 1 (0 to 1). *ND1* shows differences in many proportions, including the proportion of As (red) being added in state 3. **(C)** Example models showing the differences between tail models derived from KPAP1 WT to KPAP1 RF cell lines. Trends of note are an increase in tails that start in the primarily A-addition state 1 and a decrease in those that stay in the A-addition state 1 (1 to 1, top and bottom panels). Also, to note, is the decrease in those that stay in primarily U-addition state 2 (2 to 2, top panel only). **(D)** Example models showing the differences between tail models derived from KPAP1 WT to KPAP1 RK cell lines. Two trends are a decrease in tails that start in the primarily A-addition state 1 and an increase in those that stay in primarily U-addition state 2. **(E)** Example models showing the decrease in tails that stay in the primarily U-addition state 2 between those on pre-edited transcripts and those on early-edited transcripts. For all models, the arrow width is proportionate to the percentage of tails following the path. Proportion of color in each circle is relative to the proportion of As or Us being added in each state.

To further investigate how A and U addition differ when the KPAPs are manipulated, we used hidden Markov models (HMMs), as previously implemented to describe tail addition (Gazestani et al., 2016; Gazestani et al., 2018; Hampton et al., 2021; Smoniewski et al., 2023). We use HMMs to elucidate how the polymerases are acting during in- and ex-tail addition, as they are very sensitive to differences between libraries. For this study, we considered the two types of mitochondrial mRNA tails, in-tails and ex-tails, to define the number of nucleotide addition ‘states’ required for HMMs. In-tails result from a succession of homopolymers of As (state 1) and/or Us (state 2). In modeling, each of these states includes a small percentage of the three other nucleotides. When these are infrequent interruptions, modeling still “calls” a tail as being in the same primary state of addition. A third state (state 3) is added to HMMs expected to contain ex-tails characterized by frequent switching between As and Us. State 3 presumably models a specific state of cooperative activity of KPAP1 and KRET1, or the complexes containing these enzymes. Thus, pre-edited transcripts were modeled with state 1 and 2 only, while libraries of translation-competent transcripts, i.e. fully-edited and never-edited transcripts, were modeled with three states (**Figure 9A**).

First, we compared the HMMs between uninduced (-tet) KPAP2 OE and induced (+tet) KPAP2 OE. We found more variation between the replicates than the cell lines, which was unexpected as there was a clear difference in the A composition between the cell lines in edited *A6* tails (**Figure 3F**). We found that all *A6* edited models struggled to detect differences and define the three different states; as a result the *A6* edited model for KPAP2 does not reflect the differences shown in **Figure 3F**. To determine why this transcript was difficult to model, we randomly chose tails of 30 nt in length from each library to inspect, as tails of this length should reflect the compositional differences seen in **Figure 3F**. We found that edited *A6* tails of 30 nt were either a long in-tail or an ex-tail with a very short in-tail. Sometimes, tails started in the ex-tail state without a discernible in-tail at all. This is fundamentally different from other transcripts. This combination of vastly different tails of the same length apparently mask differences in states when used for HMMs.

Next, we compared the HMMs for uninduced (-tet) KPAP1 WT and induced (+tet) KPAP1 WT, for which some models varied widely (**Supplementary Table S6**; **Figure 9B**). For all transcripts assessed, there was an increase in tails that directly entered the primarily A-addition state 1 in cells that were overexpressing KPAP1 WT (0 to 1, **Supplementary Table S6**; **Figure 9B**). This ranged from *RPS12*e, in which the increase was as low as 2.3% in completely edited reads from the replicate 2 library, to *CYbp* and *ND1*, each with about 20% increases in both libraries analyzed. This HMM comparison revealed how KPAP1 overexpression can affect tail addition on some transcripts more than others (**Supplementary Table S6**). Specifically, transcripts with the greatest increases in tail A composition upon KPAP1 overexpression (**Supplementary Figure S6**) exhibited these features: upon KPAP1 overexpression, they possessed a higher propensity for KPAP1 rather than RET1 to initiate the tail, and RET1 bound to these transcripts upon KPAP1 overexpression was less processive. *ND1* tails in particular seemed to be very affected by KPAP1 overexpression, including an about 55% increase in the portion of As added during what normally is the A/U-addition state (**Supplementary Table S6**, column ‘3 to A’; **Figure 9B**, percentage of state 3 circle that is red in bottom panel). These effects were not detected consistently among replicates in the other transcripts. This demonstrates that while KPAP1 overexpression impacts in-tail addition (state 1 and 2) more than ex-tail addition (state 3), these effects are also transcript-specific.

We then compared models derived from the methylmimic to those derived from KPAP1 WT tails (**Supplementary Table S7**). *A6*e and *RPS12*e models had more variation between replicates than between cell lines, so no conclusions could be drawn from modeling. When compared to WT models, methylmimic models for all other transcripts showed a small decrease in tails that stayed in the A-adding state 1, which ranged from 0.2% in *CYbe* to 2.7% in *ND1* (1 to 1, **Supplementary Table S7**; **Figure 9C**). Additionally, all transcripts except *CYb* showed an increase in tails that start in the A-adding state 1 (0 to 1) and a decrease in tails that stayed in the U-adding state 2 (2 to 2, **Supplementary Table S7**; **Figure 9C**). Unlike the other transcripts, both *CYbp* and *CYbe* models showed a decrease in tails that start in state 1 in methylmimic cell lines (0 to 1, **Supplementary Table S7**; **Figure 9C**). *CYb* has an unusually high proportion of Us at the beginning of tails, which suggests that KRET1 is more likely to bind to this transcript to initiate tail addition, than for the other transcripts, and that tail addition for this transcript is slightly different than the others. Taken together, although there are general effects of KPAP1 methylmimic mutation on mRNA tails, notably, a higher likelihood that tails begin with A, some effects are transcript-specific. It was also clear that methylmimic models showed few differences from WT models in the ex-tail-adding state 3, which fits with the lack of influence of that mutation on tail length and A composition of ex-tails in general.

The hypomethyl mutant HMMs did not expose any trends that applied across all transcripts regardless of editing status (**Supplementary Table S8**). Replicates of edited transcripts were often very different from each other, which made interpretation unreliable for those models. However, there were common trends for all pre-edited and never-edited transcripts. When compared to WT models, hypomethyl mutant models showed a decrease in tails that start in the A-addition state 1 (0 to 1) and an increase in tails that remain in the state in which Us continue to be added (2 to 2, **Supplementary Table S8**; **Figure 9D**). Within these common trends, however, the magnitude of the effect was again influenced by the identity of the transcript.

Our modeling conclusions are first that overexpression of wildtype KPAP1 increases the proportion of tails that immediately start in the primarily A-adding state. Second, the methylmimic mutation of KPAP1 generally increases the proportion of tails that start in a primarily A-adding state and decreases the number of tails that remain in a U-adding state. Third, the effects of the KPAP1 hypomethyl mutation are largely the inverse of those of the methylmimic, with most transcripts showing a decrease in the proportion of tails that start in the primarily A-adding state and an increase in the number of tails that remain in the U-adding state. There are exceptions to these general trends, and the magnitude of change is transcript-specific.

Lastly, we assessed whether HMM would identify differences between pre-edited and early-edited tails for all KPAP1 and KPAP2 cell lines. Surprisingly, we found that once in the U state of addition tails are more likely to remain in that state (2 to 2) in pre-edited tails than in early-edited tails (example shown in **Figure 9E**). This suggests that if KRET1 is not replaced by KPAP1 prior to editing initiation, KRET1 will act in a less processive manner during editing. This is universally true for every cell line analyzed in this study, whether induced or not induced for KPAP1 or KPAP2 manipulation. However, the magnitude of this difference is transcript and cell line dependent; *A6* has a greater decrease (9-15%) in tails that exhibit a more processive U addition state after editing has been initiated, and *RPS12* has a smaller decrease (3-11%). Additionally, when considering the KPAP1 mutations, for *A6* the methylmimic has the lowest percentage decrease (9%), while for *RPS12* the methylmimic has the highest (11%) and the hypomethyl has the lowest (3%).

## 4 Discussion

Here we show that mitochondrial mRNA 3^′^ tail addition is affected by changing aspects of the two kinetoplastid PAP-family proteins, KPAP1 and KPAP2, in procyclic form *T. brucei*. Additionally, we present a comprehensive UTR termini analysis for five maxicircle transcripts, and elucidate the ways that A- and U-adding enzyme activity changes once editing begins on a transcript.

We detected KPAP2 occurring independently, and in protein complexes of just under 1600kDa (**Figure 2A**). The identities of these complexes are unknown, but these results may encourage the identification of KPAP2’s binding partners, which, in turn, will further define KPAP2’s function. Overexpression of KPAP2 has subtle but reproducible transcript-specific effects (**Figure 3F**). Although only one transcript was sequenced, the difference in A composition in longer tails on edited transcripts suggests that KPAP2 could play a larger role in ex-tail addition than in-tail addition. Sequencing of additional transcripts would be needed to confirm this trend.

We were able to assess KPAP1 overexpression and methylation site mutation effects in greater depth than for KPAP2 overexpression. Both silencing and overexpressing KPAP1 produced slower growth of procyclic cells suggesting that too much or too little of this vital protein is enough to disrupt cellular processes (**Figure 4E**, top row). Overexpression of WT KPAP1 increased A composition of transcript tail populations but did not greatly affect their length profiles (**Supplementary Figures S4, S6**). This supports the hypothesis that the length of tails in trypanosome mitochondria is rigidly controlled, similar to other systems (Legnini et al., 2019). However, there is no clear mechanism whereby this control would be imparted. The HMM showed that when compared to normally expressing cells, a higher proportion of transcript tails in KPAP1 overexpressing parasites began in an A-addition state (**Figure 9B**; **Supplementary Table S6**), suggesting that U tails are not simply by products of the MPsome activity (Mesitov et al., 2019; Aphasizheva et al., 2020), but are rather added in what appears to be a competitive manner between KRET1 and KPAP1.

This study is the first to explore the effects of mutation of KPAP1 arginine methylation sites on KPAP1’s role in *T. brucei*. Methylation has been shown to change the binding and function of other proteins in *T. brucei* (Goulah et al., 2006; Goulah and Read, 2007; Lott et al., 2015). Methylation could be impacting KPAP1’s RNA binding or enzymatic activity, or modifying protein binding, which could change the complexes in which KPAP1 is found. Any of these methylation effects could in turn effect tail characteristics and organism fitness. Contrary to WT KPAP1 overexpression, overexpression of KPAP1 in which both known (and closely positioned) arginine sites were mutated to a lysine (hypomethylation) or phenylalanine (methylmimic) amino acid, was not detrimental to cell growth and, in fact, could rescue the KPAP1 depletion growth phenotype (**Figures 4E**, **S2**). This suggests that these proteins are not as disruptive to cell processes when overexpressed as their WT counterpart. While many effects of mutagenesis of methylation sites on mRNA tails were transcript-specific, some trends were identified for each mutant. The methylmimic mutation sometimes resulted in more tails on *ND1* and *RPS12* transcripts suggesting that it was quicker to bind to mRNA and start adding nucleotides than WT KPAP1 (**Figure 6**). It also resulted in transcript populations with shorter tails, generally with higher adenine content at the beginning of the tails and less adenine content as the tails increased in length (**Figures 7**, **8**). This was corroborated by HMM that showed the methylmimic mutation generally resulted in an increased percentage of tails that started in the A-addition state (**Figure 9C**; **Supplementary Table S7**). Thus, KPAP1 methylation appears to simultaneously increase its likelihood of binding to mRNA, but also decrease its processivity. By contrast, while in some cases the hypomethyl mutant tails resembled the WT KPAP1 tails, some transcripts’ tails displayed lower A content at the beginning of tails (**Figure 8**) and many showed a decrease in tails that start in the primarily A-adding state (**Supplementary Table S8**; **Figure 9D**). The hypomethyl mutant also resulted in a higher percentage of *RPS12* molecules lacking tails (**Figure 6B**). Altogether, this suggests that the hypomethyl mutant may be less likely to bind to mRNA and add A tails on certain transcripts. Thus, the methylmimic and hypomethyl KPAP1 mutants generally act in opposing ways: at least with respect to KPAP1’s role in in-tails, methylation may activate this enzyme, while unmethylated KPAP1 may be less active.

The lack of differences between the mutants and the WT KPAP1 cell lines in late-edited or fully-edited tails suggests that only a subset of transcripts with specific tail characteristics continue to the late to fully-edited stages (**Figures 7**, **8**). Additionally, the fact that we rarely captured ex-tails in the fully edited libraries and infrequently captured longer tails in the late-edited transcript libraries (**Figure 7C**) is the opposite of what we were expecting. This is because tail extension is thought to be an important transitional step in fully-edited transcript processing (Aphasizheva et al., 2014). Our unexpected result is likely at least partly related to technical aspects of our PCR-based approach that favors amplification of shorter products and thus, shorter tails. However, previous data about the presence of long tails on edited transcripts comes from gels probed with sequences that could bind to mRNAs of a variety of editing states (Zhang et al., 2017). In contrast, circTAIL-seq provides convincing proof about the stage of editing for transcripts associated with each tail. Additionally, in-tails of *RPS12* edited transcripts were, in fact, shorter than those of *RPS12* pre-edited transcripts. This suggests that either only shorter in-tails progressed to ex-tails, or tails were trimmed either prior to or following ex-tailing.

Although our mutation approach for studying methylation has been used previously to mimic methylation and hypomethylation (Dillon et al., 2013; Lott et al., 2013; Kim et al., 2015), there are a few limitations. First, while the amino acid mutations mimic certain similar characteristics of arginine methylation or hypomethylation we are not able to directly manipulate arginine methylation on KPAP1 specifically and exclusively *in vivo* because the enzymes responsible for methylation all have many substrates (Lott et al., 2014). Thus, we cannot distinguish whether arginine methylation is important for the effects on tails that we observed, or that these amino acid mutations are affecting KPAP1 in other ways. Additionally, the methylmimic and hypomethylation mutants represent simplified extremes of methylation (both arginine sites methylated or not methylated for all copies of the protein) while the methylation in cells is likely more complicated and nuanced. For example, wildtype KPAP1 may have one site methylated while the other is not, or methylation could change throughout one life stage or between life stages. And lastly, differently methylated KPAP1 molecules could be associated with specific transcripts. So, while our experimental methods are accepted in the field as mimicking methylation and hypomethylation, the mutations are admittedly not a perfect model for arginine methylation and simplify the native methylation conditions that may be present in the cell.

Regardless of manipulations to either KPAP, we found that there are changes in the way tail-adding enzymes function prior to editing and after editing has begun (**Figure 9E**). Pre-edited transcript tails were more likely to remain in the state in which Us continue to be added (state 2) than early-edited tails. This suggests that once editing has begun, tails move from U-addition back to A-addition more readily, possibly describing the competition between KRET1 and KPAP1 during tailing.

This study is designed to identify potential mechanisms of tail addition regulation, and as such it did provide multiple clues described above. However, as a side benefit, it represents one of the most comprehensive UTR termini assessments in *T. brucei* to date (**Figure 5**). Other studies have determined UTR termini for kinetoplast genes (Gazestani et al., 2018; Sement et al., 2018), but these did not include both the pre-edited and edited versions of transcripts. More comprehensive analysis could provide clues to pre-mRNA processing events that are still not completely understood in *T. brucei* such as transcription initiation or potential endonucleolytic cleavage events. We found that for each transcript sequenced, termini positions were remarkably consistent between cell lines and replicates, indicating that termini processing does not allow for much variation.

Our alignment methodology has been carefully assessed for accurate termini and minimization of incorrect alignments, especially for transcripts that are edited. We also identified extensive editing in the 3^′^ UTR for *A6* and the 5^′^ UTR of *RPS12*, adding about 25 Us and 15 Us, respectively, to the pre-edited sequence. This demonstrates just how extensively editing can progress into transcripts’ non-coding regions. The patterns of completed editing were generally consistent, suggesting that specific templates (gRNAs) exist that are dedicated to these patterns. In turn, this suggests that 3’ UTR edited sequences may be important for RNA regulation or protein binding. Additionally, from these data, a striking trend of similar 5^′^ UTR lengths suggests that the length of the 5^′^ UTR is either determined by similar mechanisms acting on most transcripts, or is conserved for a specific reason. *ND1* was the only transcript that had highly variable termini and is the only transcript that we sequenced that does not have another gene near to it upstream. While mitochondrial transcription in trypanosomes is still not completely understood and may include examples of polycistronic products which may require subsequent endonucleolyic cleavage, there is also evidence of monocistronic transcription from unique start sites (Sement et al., 2018). The two distinct trends in UTR termini may be because transcription is achieved through a mix of these processes.

In summary, we have shown that both KPAPs affect tail characteristics in a transcript and editing-status specific manner. KPAP2 is found in a complex and while its effects on tails may seem minimal, they are measurable and reproducible. Mutations to KPAP1 arginine methylation sites suggest that methylation may increase KPAP1’s ability to bind to mRNA but reduce its processivity. Additionally, we were able to show that tail adding enzymes change their behavior once editing begins, and comprehensively evaluate UTR termini on five transcripts. This work furthers our understanding of tail addition in trypanosome mitochondria and transcript-specific tail characteristics.

## Data availability statement

The datasets presented in this study can be found in online repositories. The names of the repository/repositories and accession number(s) can be found below: https://www.ncbi.nlm.nih.gov/, PRJNA938451.

## Author contributions

CS: Conceptualization, Data curation, Formal Analysis, Investigation, Methodology, Visualization, Writing – original draft, Writing – review & editing. PM: Data curation, Methodology, Writing – review & editing. MH: Data curation, Visualization, Writing – review & editing. AP: Formal Analysis, Writing – review & editing. SF: Formal Analysis, Writing – review & editing. RS: Funding acquisition, Project administration, Resources, Supervision, Writing – review & editing. SZ: Conceptualization, Funding acquisition, Investigation, Methodology, Project administration, Resources, Supervision, Writing – review & editing.
