# Supplementary material for: Manipulation of mitochondrial poly(A) polymerase family proteins in Trypanosoma brucei impacts mRNA termini processing
Source: Front Parasitol. 2024 Jan 11;2:1298561. doi: 10.3389/fpara.2023.1298561 (PMC11732105; doi:10.3389/fpara.2023.1298561)
Supplement: Supplementary file 1 [file DataSheet_1.pdf]

Supplementary Material

1 SUPPLEMENTARY TABLES AND FIGURES

1.1 Figures

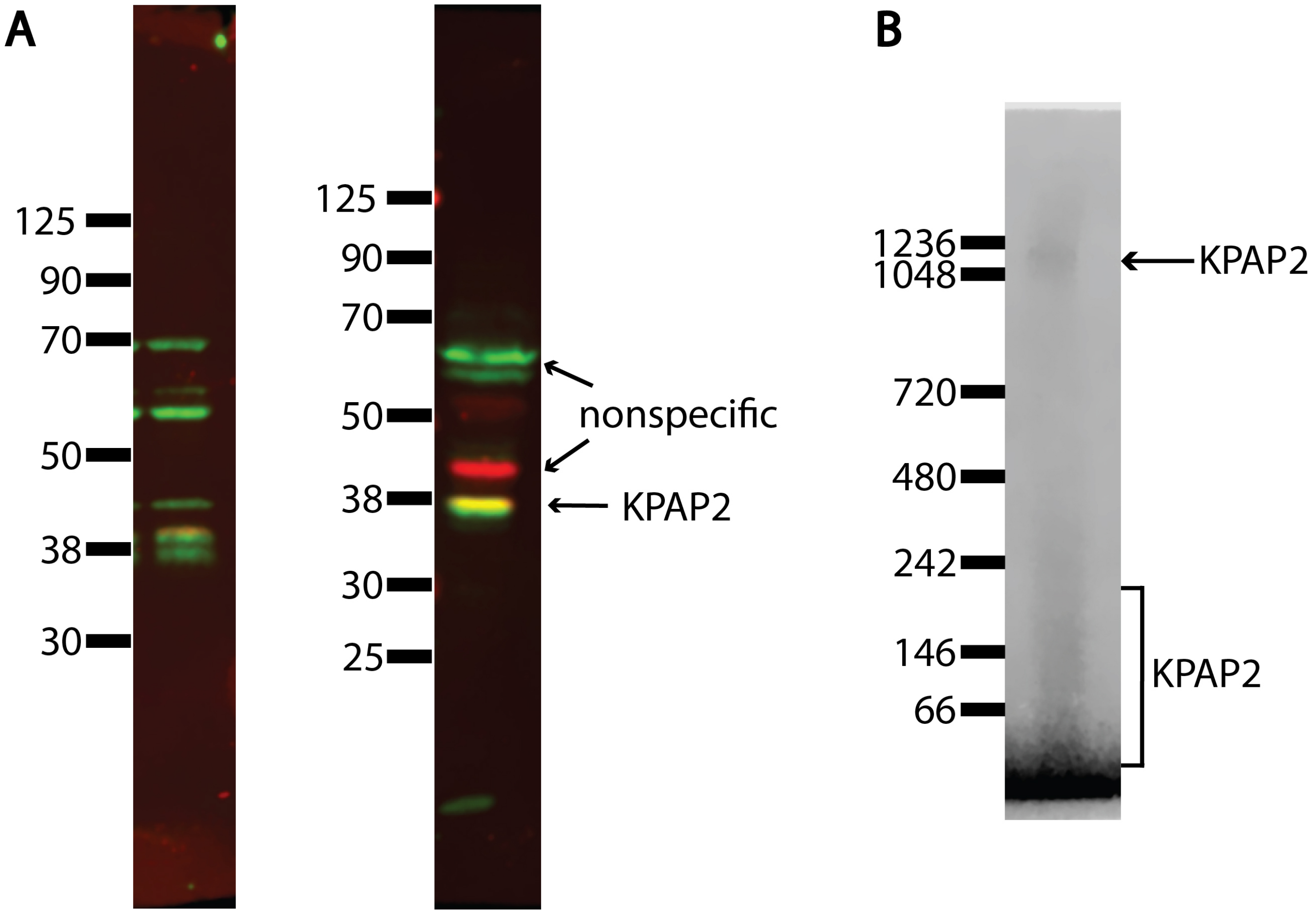

Figure S1. Detection of KPAP2. (A) KPAP2 OE protein extract probed in an immunoblot with histidine and KPAP2 antibodies before purification (left) and after KPAP2 antibody purification (right). Anti-histidine, red; anti-KPAP2, green. Yellow is where anti-histidine and anti-KPAP2 both bind. (B) Blue native polyacrylamide gel electrophoresis followed by immunoblot of protein from the KPAP2 OE cell line. Purified KPAP2 antibody was used to detect KPAP2.

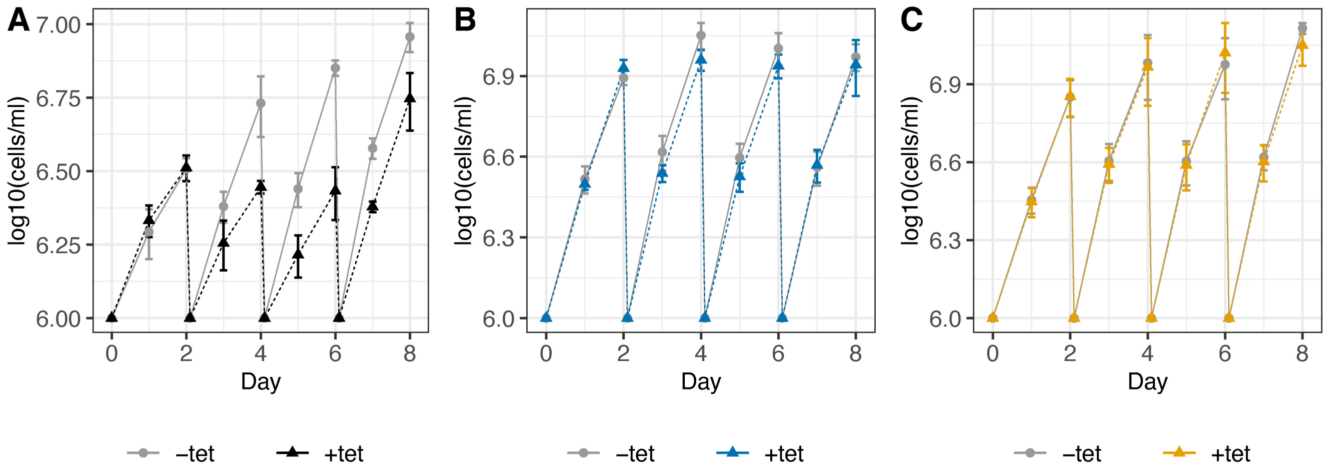

Figure S2. Cell growth is negatively affected by WT KPAP1 overexpression, but not by mutant KPAP1 overexpression. (A) Growth curves of KPAP1 OEWT cell line in which, when induced, an exogenous wildtype version of KPAP1 is overexpressed. (C) Growth curve of KPAP1 OERF (methylmimic) cell line in which, when induced, an exogenous mutated version of KPAP1 is overexpressed. (D) Growth curve of KPAP1 OERK (hypomethyl) cell line in which, when induced, an exogenous mutated version of KPAP1 is overexpressed. Error bars represent standard deviation. Cells are kept in log phase growth by diluting them every two days to 1x10^6 cells/ml. Experiments were done in triplicate. Uninduced, -tet; induced, +tet.

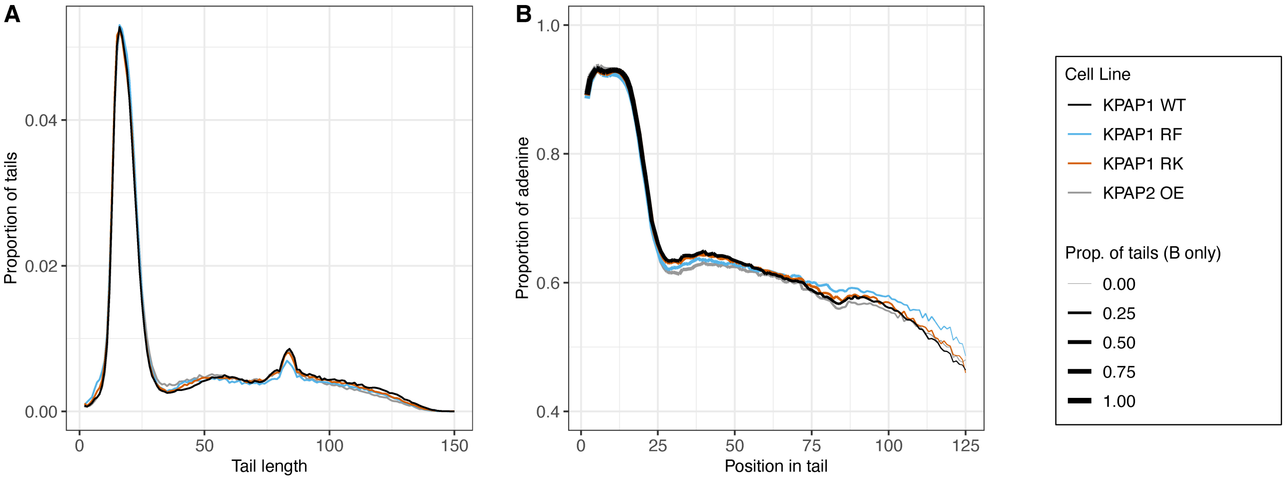

Figure S3. When uninduced, all cell lines have similar tail characteristics for *Trypanosoma brucei* mitochondrial transcript *CO1*. (A) Tail length population density curves for the *CO1* transcript. (B) Population density curves of aggregate proportions of nucleotides that are adenine at each position along each tail for the *CO1* transcript. The thickness of the line represents the proportion of the tail population that is long enough to contribute to the data at each nucleotide position. As lines become thinner, adenine content data is supported by fewer total tails because very few long tails are represented in the populations.

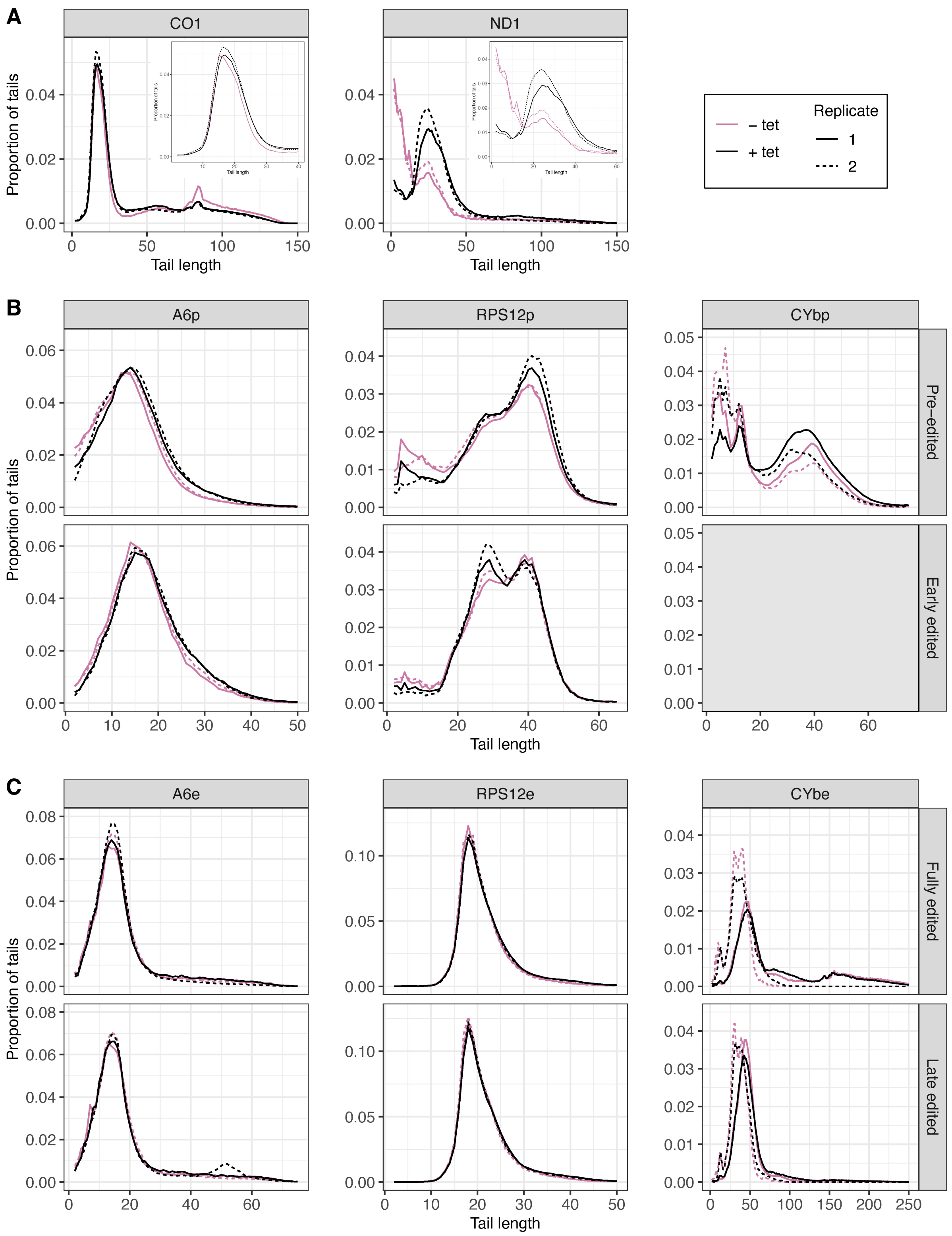

Figure S4. Impacts of overexpression of WT KPAP1 on mRNA tail length. (A) Population density curves of tail lengths for never-edited transcripts *CO1* and *ND1*. Inset graph is zoomed-in on the length of the majority of the tails, which are shorter (less than 60 nt) than the longest tails within the total tails sequenced. There is only one replicate for -tet *CO1* as noted in the methods. (B) Population density curves of tail lengths for pre-edited and early edited transcripts *A6*, *RPS12*, and *CYb*. Grey box for the early-edited *CYbp* tails indicates there were too few tails to analyze. (C) Population density curves of tail lengths for fully edited and late edited transcripts *A6*, *RPS12*, and *CYb*. Reads are derived from primers designed to bind to either pre-edited transcripts (p) or edited transcripts (e).

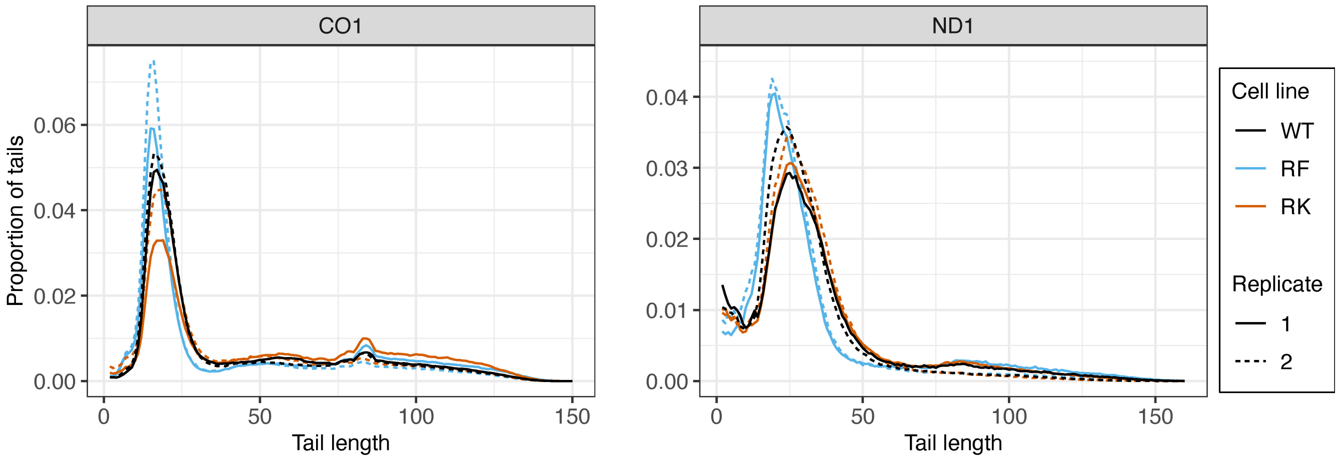

Figure S5. Mutations at the arginine methylations sites on KPAP1 do not affect the longer tails on never-edited transcripts *CO1* and *ND1*. Population density curves of all tails for never-edited transcripts *CO1* and *ND1*.

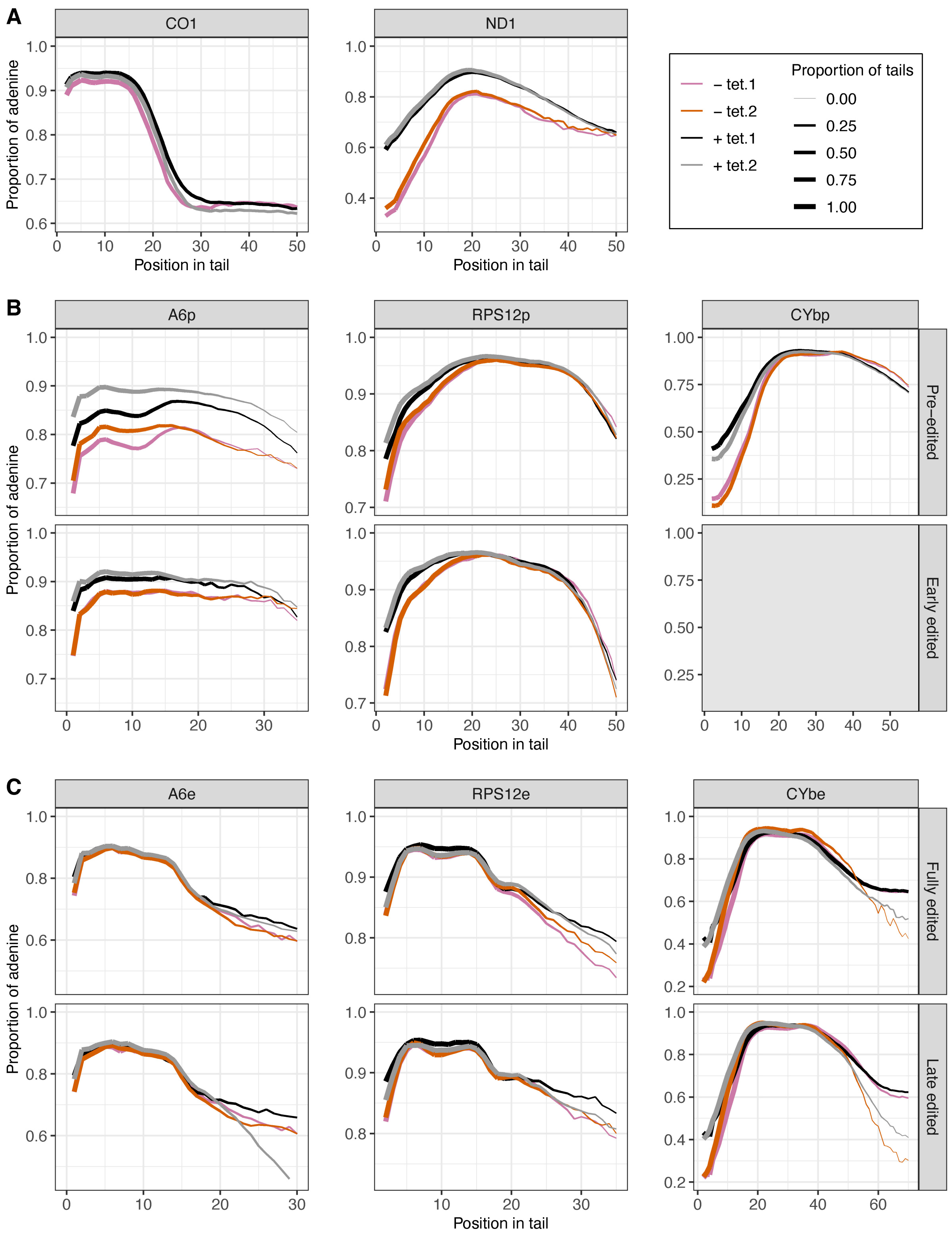

Figure S6. Overexpression of WT KPAP1 affects the adenine composition of tails of never-edited and pre-edited transcripts. (A) Population density curves of proportion of adenine along tail for never-edited transcripts *CO1* and *ND1* zoomed in on the more abundant shorter tails, which are less than 60nt. There is only one replicate for -tet *CO1* as noted in the methods. (B) Population density curves of proportion of adenine along tail for pre-edited and early edited transcripts *A6*, *RPS12*, and *CYb*. Grey box for the early-edited *CYb*p tails indicates there were too few tails to analyze. (C) Population density curves of proportion of adenine along tail for fully edited and late edited transcripts *A6*, *RPS12*, and *CYb*. Reads are derived from primers designed to bind to either pre-edited transcripts (p) or edited transcripts (e).

1.2 Tables

Table S1. Primer and gBlock sequences used to construct and validate cell lines.

Table S2. PCR conditions and primers used. Lowercase indicates Ts (As in reverse complement) that are added during editing. Blue, Illumina 3′ adapter sequence; black lowercase, Illumina 5′ adapter sequence; bolded red, barcode; red Ns, variable sequence; bolded black uppercase, gene specific sequence. Primers were newly designed unless otherwise noted. # (Gazestani et al. 2016), * (Mesitove et al., 2019), ˆ (Kao and Read, 2007).

Table S3. Number of raw reads and reads used in analysis. The number of reads used in the analysis only includes those that aligned to the UTR templates. Top group of *CO1* reads were only used for control analysis presented in Figure S3.

Table S4. Diversity metrics for each library used in downstream analysis. Unless otherwise noted, all reads were considered, even those that did not align to the DNA template. Q1, 1st quartile; Q2, 2nd quartile; Q3, 3rd quartile.

Table S5. Reads broken into partially edited and fully pre-edited or fully-edited. ”Full” indicates that no editing was initiated for pre-edited transcripts and editing was completed correctly through the coding region of edited transcripts.

Transcript	Replicate	Editing status	Changes in percentages of tails moving from state *n* to state *n*				Changes in percentages of adenines added in each state (proportion of red in circles)		
			0 to 1	1 to 1	2 to 2	3 to 3	1 to A	2 to A	3 to A
A6p	Rep 1	fully-pre	9.2	1.4	0.7	NA	0.2	-0.2	NA
		partial	9.5	1	0	NA	0	0.6	NA
	Rep 2	fully-pre	12.6	1.5	-0.4	NA	0.2	-0.1	NA
		partial	11.6	0.8	-1.2	NA	-0.3	1.6	NA
RPS12p	Rep 1	fully-pre	10	0.5	1.6	NA	0	-0.7	NA
		partial	13.7	0.4	-0.1	NA	-0.1	-1.5	NA
	Rep 2	fully-pre	10	0.6	1.2	NA	0	-0.7	NA
		partial	16	0.4	-2	NA	0	0.2	NA
CYbp	Rep 1	fully-pre	20.5	-0.2	-2.8	NA	1.5	1.2	NA
	Rep 2	fully-pre	18.4	-0.9	-2.3	NA	1.4	1.6	NA
A6e	Rep 1	complete	5.5	0.6	-9.0	1.2	0.0	-2.5	0.7
		partial	5.3	0.7	-5.0	1.2	0.1	-3.5	1.1
	Rep 2	complete	2.9	-0.5	-0.6	-4.0	0.3	2.2	4.6
		partial	18.9	1.8	-26.0	4.2	-2.1	-29.4	-8.6
RPS12e	Rep 1	complete	6.3	0.6	2.9	-0.5	-0.2	0.0	2.8
		partial	8.5	0.9	1.4	0.2	-0.4	-0.3	1.1
	Rep 2	complete	2.3	0.4	2.2	1.3	-0.2	0.1	-0.5
		partial	4.7	0.6	2.4	0.5	-0.3	0.5	-0.5
CYbe	Rep 1	complete	15.4	0.0	-1.4	-0.2	1.1	0.4	0.1
		partial	16.4	0.0	-1.5	-0.2	1.0	0.4	0.2
	Rep 2	complete	13.0	0.0	-1.1	8.1	0.3	0.6	3.8
		partial	14.0	0.4	-1.6	13.0	0.2	0.6	9.7
CO1	Rep 1	NA	5.7	0.8	5.0	-0.3	0.5	-0.7	0.0
ND1	Rep 1	NA	19.7	-1.9	9.0	23.3	16.5	6.1	55.8
	Rep 2	NA	20.3	-1.7	8.5	22.6	13.8	6.9	54.1

Table S6. Hidden Markov modelling shows differences in tail addition enzyme activity when KPAP1 is overexpressed. Shown are the differences in percentages between models derived from induced KPAP1 OE cell lines compared to models derived from uninduced KPAP1 OE cell lines. Headers refer to visual models displayed in Figure 13. Positive numbers (red shades) denote an increase in the induced cell line and negative numbers (blue shades) denote a decrease in the induced cell line.

Transcript	Replicate	Editing status	Changes in percentages of tails moving from state *n* to state *n*				Changes in percentages of adenines added in each state (proportion of red in circles)		
			0 to 1	1 to 1	2 to 2	3 to 3	1 to A	2 to A	3 to A
A6p	Rep 1	fully-pre	10.1	-1.5	-9.3	NA	0.1	2.1	NA
		partial	5	-1.5	-5.9	NA	-0.2	2.9	NA
	Rep 2	fully-pre	3.8	-1.2	-3.8	NA	0.2	0.8	NA
		partial	2.5	-1.2	-1.7	NA	0	0.9	NA
RPS12p	Rep 1	fully-pre	11.6	-0.7	-5.5	NA	-0.3	1.5	NA
		partial	7.8	-0.8	-8.4	NA	0	3.8	NA
	Rep 2	fully-pre	9.3	-0.7	-2.7	NA	-0.1	-0.1	NA
		partial	8	-0.7	-5.6	NA	0.1	0.6	NA
CYbp	Rep 1	fully-pre	-8.9	-1.5	0	NA	0	1	NA
	Rep 2	fully-pre	-5.2	-1.6	0.1	NA	-0.1	0.5	NA
A6e	Rep 1	complete	-6.7	-0.7	7.2	0.5	0.3	12.6	1.5
		partial	-5.4	-0.3	-1.0	1.3	0.0	10.3	1.6
	Rep 2	complete	-1.5	0.7	-1.3	7.7	-0.3	52.5	-8.5
		partial	3.2	-0.3	-7.7	0.9	0.4	-0.4	3.2
RPS12e	Rep 1	complete	-1.7	-0.9	-2.0	0.1	0.5	0.5	0.3
		partial	-0.9	-0.5	0.4	-1.0	0.5	1.1	-1.1
	Rep 2	complete	5.4	0.6	1.4	-1.8	0.3	-0.6	-2.6
		partial	5.5	0.6	0.4	-1.6	0.4	-0.3	-1.9
CYbe	Rep 1	complete	-6.8	-0.2	-0.4	-3.3	0.7	0.6	0.5
		partial	-7.4	-0.4	-0.3	-9.1	1.1	1.0	4.1
	Rep 2	complete	-3.4	-0.4	0.3	0.8	0.5	0.6	3.0
		partial	-4.0	-0.4	0.3	-0.8	0.7	0.6	17.1
CO1	Rep 1	NA	0.9	-1.1	-3.4	0.2	-0.2	-0.9	-0.5
	Rep 2	NA	3.8	-0.8	-3.0	-0.1	0.3	1.0	2.6
ND1	Rep 1	NA	6.2	-2.7	-2.2	0.1	-0.6	0.3	-0.4
	Rep 2	NA	4.2	-2.2	-2.2	0.1	-0.1	-0.8	2.9

Table S7. Hidden Markov modelling shows differences in tail addition enzyme activity of WT KPAP1 compared to methylmimic KPAP1. Shown are the differences in percentages between models derived from WT KPAP1 cell lines compared to models derived from methylmimic (RF) KPAP1 cell lines. Headers refer to visual models displayed in Figure 13. Positive numbers (red shades) denote an increase in the mutant cell line and negative numbers (blue shades) denote a decrease in the mutant cell line.

			Changes in percentages of tails moving from state *n* to state *n*				Changes in percentages of adenines added in each state (proportion of red in circles)		
**Transcript**	**Replicate**	**Editing status**	**0 to 1**	**1 to 1**	**2 to 2**	**3 to 3**	**1 to A**	**2 to A**	**3 to A**
A6p	Rep 1	fully-pre	-4.5	0.1	2.5	NA	-0.5	0.1	NA
		partial	0.1	0.2	4.5	NA	-0.5	0.7	NA
	Rep 2	fully-pre	-8.2	0.5	6.9	NA	-0.6	-1.4	NA
		partial	-1	0.5	4.8	NA	-0.3	-1.2	NA
RPS12p	Rep 1	fully-pre	-5.8	-0.4	2.4	NA	-0.4	-1.5	NA
		partial	-8.7	-0.2	8	NA	-0.4	-0.5	NA
	Rep 2	fully-pre	-7.8	-0.2	5.5	NA	-0.3	-2.6	NA
		partial	-7.9	0	11.2	NA	-0.4	-3.8	NA
CYbp	Rep 1	fully-pre	-6	-0.1	0.4	NA	-0.3	0.6	NA
	Rep 2	fully-pre	-4	0.3	0.4	NA	-0.4	0	NA
A6e	Rep 1	complete	-0.1	0.5	-8.3	0.8	-0.3	-4.5	2.6
		partial	6.1	0.0	3.2	1.9	-0.4	-28.8	-1.4
	Rep 2	complete	2.4	1.5	-5.3	7.9	-0.8	51.7	-10.5
		partial	-5.0	-0.7	12.8	-2.3	1.7	29.7	11.3
RPS12e	Rep 1	complete	2.4	-0.2	-2.6	-1.6	0.4	0.7	0.5
		partial	1.3	-0.2	-2.6	-1.0	0.4	0.8	0.0
	Rep 2	complete	4.9	1.4	2.7	-2.5	0.0	0.4	-6.0
		partial	5.0	1.4	0.9	-3.1	0.0	-1.7	-3.7
CYbe	Rep 1	complete	1.4	-0.1	-1.4	-13.1	0.7	1.1	0.6
		partial	0.3	-0.5	-0.8	-21.8	1.1	1.6	7.0
	Rep 2	complete	-2.0	-0.2	0.1	1.8	-0.4	0.8	3.6
		partial	-1.7	0.2	-0.1	1.6	-0.1	0.8	13.1
CO1	Rep 1	NA	-0.6	-0.1	2.5	0.2	-0.2	0.5	0.8
	Rep 2	NA	-1.1	0.2	4.4	-0.2	0.2	0.8	1.8
ND1	Rep 1	NA	-1.5	-0.1	0.6	-0.1	-0.6	2.3	0.4
	Rep 2	NA	-2.4	0.4	0.5	-0.3	-0.4	1.5	1.8

Table S8. Hidden Markov modelling shows differences in tail addition enzyme activity of WT KPAP1 compared to hypomethyl mutant KPAP1. Shown are the differences in percentages between models derived from WT KPAP1 cell lines compared to models derived from hypomethyl mutant (RK) KPAP1 cell lines. Headers refer to visual models displayed in Figure 13. Positive numbers (red shades) denote an increase in the mutant cell line and negative numbers (blue shades) denote a decrease in the mutant cell line.
